# CDK1 Is a Synthetic Lethal Target for KRAS Mutant Tumours

**DOI:** 10.1371/journal.pone.0149099

**Published:** 2016-02-16

**Authors:** Sara Costa-Cabral, Rachel Brough, Asha Konde, Marieke Aarts, James Campbell, Eliana Marinari, Jenna Riffell, Alberto Bardelli, Christopher Torrance, Christopher J. Lord, Alan Ashworth

**Affiliations:** 1 The CRUK Gene Function Laboratory, The Institute of Cancer Research, London, SW3 6JB, United Kingdom; 2 Breakthrough Breast Cancer Research Centre, The Institute of Cancer Research, London, SW3 6JB, United Kingdom; 3 IFOM—FIRC Institute of Molecular Oncology, Via Adamello 16, 20139, Milan, Italy; 4 Horizon Discovery, 7100 Cambridge Research Park, Waterbeach, Cambridge, CB25 9TL, United Kingdom; University of South Alabama Mitchell Cancer Institute, UNITED STATES

## Abstract

Activating KRAS mutations are found in approximately 20% of human cancers but no RAS-directed therapies are currently available. Here we describe a novel, robust, KRAS synthetic lethal interaction with the cyclin dependent kinase, CDK1. This was discovered using parallel siRNA screens in KRAS mutant and wild type colorectal isogenic tumour cells and subsequently validated in a genetically diverse panel of 26 colorectal and pancreatic tumour cell models. This established that the KRAS/CDK1 synthetic lethality applies in tumour cells with either amino acid position 12 (p.G12V, pG12D, p.G12S) or amino acid position 13 (p.G13D) KRAS mutations and can also be replicated *in vivo* in a xenograft model using a small molecule CDK1 inhibitor. Mechanistically, CDK1 inhibition caused a reduction in the S-phase fraction of KRAS mutant cells, an effect also characterised by modulation of Rb, a master control of the G_1_/S checkpoint. Taken together, these observations suggest that the KRAS/CDK1 interaction is a robust synthetic lethal effect worthy of further investigation.

## Introduction

KRAS, also known as the Kirsten rat sarcoma viral oncogene homolog protein (V-Ki-ras2), is a member of the RAS superfamily [1, 2]. RAS proteins (HRAS, KRAS and NRAS) are small GTPases that cycle between inactive guanosine diphosphate (GDP)-bound and active guanosine triphosphate (GTP)-bound conformations. RAS activity regulates a complex signalling network including the RAF-MEK-ERK cascade, the phosphatidylinositol 3-kinase (PI3K) pathway and the effector family of exchange factors for the RAL small GTPases [3–5]. Through the combined action of these signalling pathways, expression of activated mutant RAS is thought to promote several of the characteristics of malignant transformation. The *KRAS* oncogene is one of the most frequently mutated genes in human cancer [6], being altered in approximately 20% of all human tumours [7]. Oncogenic forms of *KRAS* have profound effects on signalling, which can result in a hyper-proliferative and anti-apoptotic phenotype [3, 8–10]. In addition, *KRAS* mutations affecting amino acid position p.G12, cause resistance to EGFR targeted therapy in colorectal cancer (CRC) [11, 12].

Because of the frequency of *KRAS* mutations in human cancers considerable attention has been paid to targeting this oncogene. These efforts include; (i) approaches that are based on inhibiting signal transduction pathways that act downstream of KRAS, such as the use of MEK inhibitors [13], (ii) the identification of synthetic lethal (SL) interactions with mutant KRAS [14–21] and, (iii) direct small molecule inhibition of KRAS, an approach that exploits the presence of a mutant cysteine residue in KRAS mutant tumour cells with p.G12C mutations [22]. In the case of the SL approaches to targeting mutant KRAS, a considerable challenge has been in discriminating those KRAS SL effects that are readily abrogated by other genetic/ epigenetic changes in the tumour cell (soft SL effects) from those that are more resilient to these changes (hard SL effects) [23].

Here, we describe the identification of a novel KRAS SL interaction involving the cyclin dependent kinase, CDK1. This was identified using siRNA screens, was shown to operate in a genetically diverse set of colorectal and pancreatic tumour cell models and was replicated with small molecule inhibitors of CDK1, both *in vitro* and *in vivo*.

## Materials and Methods

### Cell lines

The LIM1215 and SW48 KRAS isogenic cell lines were generated and provided by Horizon Discovery. The LIM1215 cell lines were cultured in RPMI supplemented with 10% FBS and SW48 cell lines were grown in DMEM with 10% FBS. The C2BBe1, HT115, HT29, HT55, LS411N, RKO, SNU-C1, SW1417, WiDr, DLD1, GP2D, HCT116, LOVO, LS513, SKCO1, SW1116, SW480, SW620, T84, BxPC-3, HPAC, MIAPACA2, PANC1, PL45 and PL5 were obtained from the American Type Tissue Collection and cultured according to the suppliers’ instructions. All cell lines were routinely confirmed as being mycoplasma negative using the MycoAlert Kit (Lonza) throughout experimentation.

### Reagents

AZD5438, RO-3306, AT7519, Dinaciclib and PD023309 were purchased from Selleck chemicals. Antibodies targeting pan-RAS (Millipore), KRAS (Sigma), β-Actin, PARP-1 (F-2) (Santa Cruz), CDK1, Phospho-CDK1 (Thr161), Rb and Phospho-Rb (Ser 807/811) (Cell Signaling Technology), were employed in western blot.

### High-throughput siRNA screening

A 384 well plate arrayed siRNA library targeting 784 genes (Dharmacon) was used (gene list described in S1 Table). Each well contained a SMARTPool of four distinct siRNA species targeting different sequences of the target transcript. Additional positive (siPLK1) and negative (siCON1, siCON2 and AllStar (Dharmacon and Qiagen, respectively)) controls were also added to each plate. The LIM1215 KRAS isogenic cell lines were plated in nine replica plates at a density of 500 cells per well and reverse transfected using RNAiMax (Life Technologies). After seven days cell viability in each well was estimated using a CellTitre-Glo assay. Data was processed as described in [24]. Each screen was performed in triplicate. The luminescence value from each well on each plate was first log_2_ transformed. To account for plate-to-plate variation, we calculated the median effect in each plate and then normalized each well value according to the plate median. Plate normalized values from triplicate screens were then combined by the calculation of median values. To allow cell inhibitory effects to be compared between cell lines and screens, we converted median plate normalized values to Z, or standardized, scores, using the calculation Z_a_ = (x_a_−screen median) / variance of screen, where Z_a_ = Z score for gene *a*, x_a_ = plate normalized value for gene *a*. The screen median was calculated according to the median value for all 784 siRNAs in the screen and the variance of the screen was estimated by calculation of the Median Absolute Deviation (MAD).

### High throughput screen siRNA revalidation

Revalidation of the siRNA screen hits was performed by deconvolution of the SMARTpool into four distinct oligonucleotide species targeting four different sequences of the gene. Reverse transfection and viability analysis were performed as described above. RNAi gene silencing was also validated by western blot, where the cells were plated in 6-well plates and collected 48 hours after transfection for preparation of protein lysates and western blot analysis.

### Protein analysis

Cells were lysed, electrophoresed and immunoblotted as described previously [25].

### Cell cycle analysis

For cell cycle analysis by DNA content, cells were plated in 10 cm dishes and exposed to the drug/siRNA in study. After the drug/siRNA exposure, cells were harvested, washed with PBS, and fixed in 70% (v/v) ethanol. Cells were then washed twice in PBS and resuspended in 1 mL of PBS containing 100 μL RNase A (Sigma) and 20 μL propidium iodide (PI) (Sigma). Total DNA content was quantified and analyzed by flow cytometry on a Becton Dickinson fluorescence-activated cell scan cytometer and data was analysed using BD FACSDiva Software (BD Biosciences).

### EdU Cell Proliferation Assay

The Click-iT EdU Alexa Fluor 647 Flow Cytometry Assay Kit was used for analyzing DNA replication in proliferating cells, according to the manufacturer’s instructions. EdU (5-ethynyl-2 ′-deoxyuridine) is a thymidine analog, which is incorporated into DNA during active DNA synthesis.

Cells were incubated with 10 μL of a 10 mM solution of EdU in a 10 cm dish for 1h at 37°C, 5% CO2. After the incubation the cells were harvested and washed once with 3 mL of 1% (w/v) BSA in PBS. The cells were then centrifuged and incubated with 100 μL of Click-iT fixative for 15 minutes at room temperature, protected from light. The cells were washed with 3 mL of 1% BSA (w/v) in PBS, centrifuged, then resuspended in 100 μL of 1X Click-iT saponin-based permeabilization and wash reagent and incubated for 15 minutes. 0.5 mL of Click-iT reaction cocktail was added to each tube and incubated for 30 minutes at room temperature, protected from light. The cells were washed once with 3 mL of 1X Click-iT saponin-based permeabilization and wash reagent, centrifuged and resuspended in 500 μL of 1X Click-iT saponin-based permeabilization and wash reagent. DAPI staining was added for DNA staining. Standard flow cytometry methods were used for determining the percentage of S- phase cells in the population and DNA content.

### DNA sequencing

Genomic DNA was isolated from the cell lines using the Puregene—blood, cell and tissue kit (Qiagen), according to manufacturer’s instructions, eluted in 20 μL H2O and stored at -20°C. DNA concentration was measured using a spectrophotometer measuring the UV absorbance at 260 nm. Typically PCR reactions contained 10–100 ng DNA, and were done in 50 μL reaction volume, using Expand High Fidelity PCR system (Roche). PCR conditions were as follows: 95°C for 5 min; thirty cycles of 94°C for 1 min, 55°C for 1 min, 72°C for 45 sec, followed by a single cycle at 72°C for 5 min and 4°C thereafter. PCR products were purified from agarose gel using QIAquick Gel extraction Kit (Qiagen). Cycle sequencing was carried out using BigDye Terminator v3.1 Cycle Sequencing kit (Applied Biosystems, Foster City, CA) with an initial denaturation at 95°C for 1 min, then 25 cycles at 96°C for 10 sec, 50°C for 5 sec, and single cycle at 60°C for 4 min. Sequencing products were purified using DyeEx 2.0 spin protocol for Dye-Terminator removal (Qiagen), and analysed using the ABI PRISM 377 DNA sequencer (Perkin Elmer). The data was analysed using Sequencher 4.8 software.

### Cell viability assays

Cells were plated in 96-well (250–1000 cells/well (depending on the cell line)) plates in 80 μL. After 24 hours, drug or vehicle (DMSO) dilutions in media were added to the cells to make a total volume of 100 μL. Cells were left exposed to drug for five days. Cell viability was assessed using the luminescent CellTiter-Glo reagent (Promega) whereby 50 μL of reagent diluted 1:4 in PBS was added to each well, which was shaken for 10 minutes at room temperature according to the manufacturer’s protocol. Luminescence was measured using the Victor X5 Multilabel plate reader (Perkin Elmer).

### RAS activation assay

Activated RAS was measured using a RAS activation assay kit according to manufacturer’s instructions (Millipore). RAS alternates between an active, GTP-bound state and an inactive, GDP-bound state. RAS family effector proteins specifically recognize the GTP-bound form of RAS. This is exploited experimentally to determine the levels of RAS protein activation. The assay uses the RAS-binding domain (RBD) of the RAS effector kinase c-RAF, which binds specifically to the GTP-bound form of RAS proteins. The RAF-RBD is conjugated with agarose beads, which allows the precipitation of the RAF-RBD/GTP-RAS complex. For the RAS pull-down assay, 500 μg of whole cell lysates (freshly collected) were incubated with the RAF-RBD-agarose beads for 45 min at 4oC with gentle agitation. After washing the agarose beads, the amount of GTP-RAS was quantified by western blotting of purified samples with a mouse monoclonal antibody recognizing all three isoforms of RAS. MCF7 and HeLa cells were used as controls of the experiment. HeLa cells were also stimulated with 50 nM EGF as a positive control.

### *In vivo* experiments

All mouse work was carried out in accordance with the Institute of Cancer Research (ICR) guidelines and with the UK Animals (Scientific Procedures) Act 1986 and approved by the ICR Animal Welfare and Ethical Review Body. Animals were housed in IVC type cages (Optimouse–Animal Care Systems Inc.), which were maintained under negative airflow. Mice were companion held and a density commensurate with the UK Home Office Code of Practice for the Housing and Care of Animals Bred, Supplied or Used for Scientific Purposes. Animals were provided with Corncob bedding, nesting material and environment enrichment. All animals were fed Ad-libitum with Lab diet 5002 rodent diet. Water was filtered and chlorinated. Animal holding rooms were maintained within the parameters recommended in the Home Office Code of Practice with temperatures being 21°C +/- 2 degrees, Humidity 55% +/- 10% and a light cycle of 12 hours dark/light. Animals were monitored daily by facility staff for basic husbandry requirements and signs of ill health. Study animals were also monitored by AK and SCC.

For assessment of the *in vivo* efficacy of AZD5438, 5x10^6^ of SW620 cells, or SW48 KRAS WT or p.G12V isogenic cells were injected into the flank regions of female athymic Balb/C mice, twenty mice per cell line (Harlan Laboratories). In the drug arm ten mice were treated once daily with AZD5438 by oral administration starting immediately after tumour establishment at a dose of 20mg/kg and ten mice were treated once daily with vehicle (0.5% methylcellulose) in the control arm.

Tumour growth was monitored at least twice a week, taking two-dimensional measurements with callipers. The mice were monitored daily for toxicity, and culled when the maximum tumour volume was reached (1.2 cm^3^) or if there was more than 20% weight loss. Tumour volume was calculated using the formula (L*W)*(SQRT(L*W))*(π/6) (L, length, W, width, π, pi), where length represented the longer diameter of an asymmetrical tumour.

All procedures were performed according to the project licence number 70/6367 and under the regulations of the Animals (Scientific Procedures) Act 1986. Mice were sacrificed by the Schedule 1 methods of cervical dislocation or decapitation according to the UK Animals Act 1986.

## Results

### Synthetic lethal screening in an isogenic KRAS mutant tumour cell system

**Fig 1:**
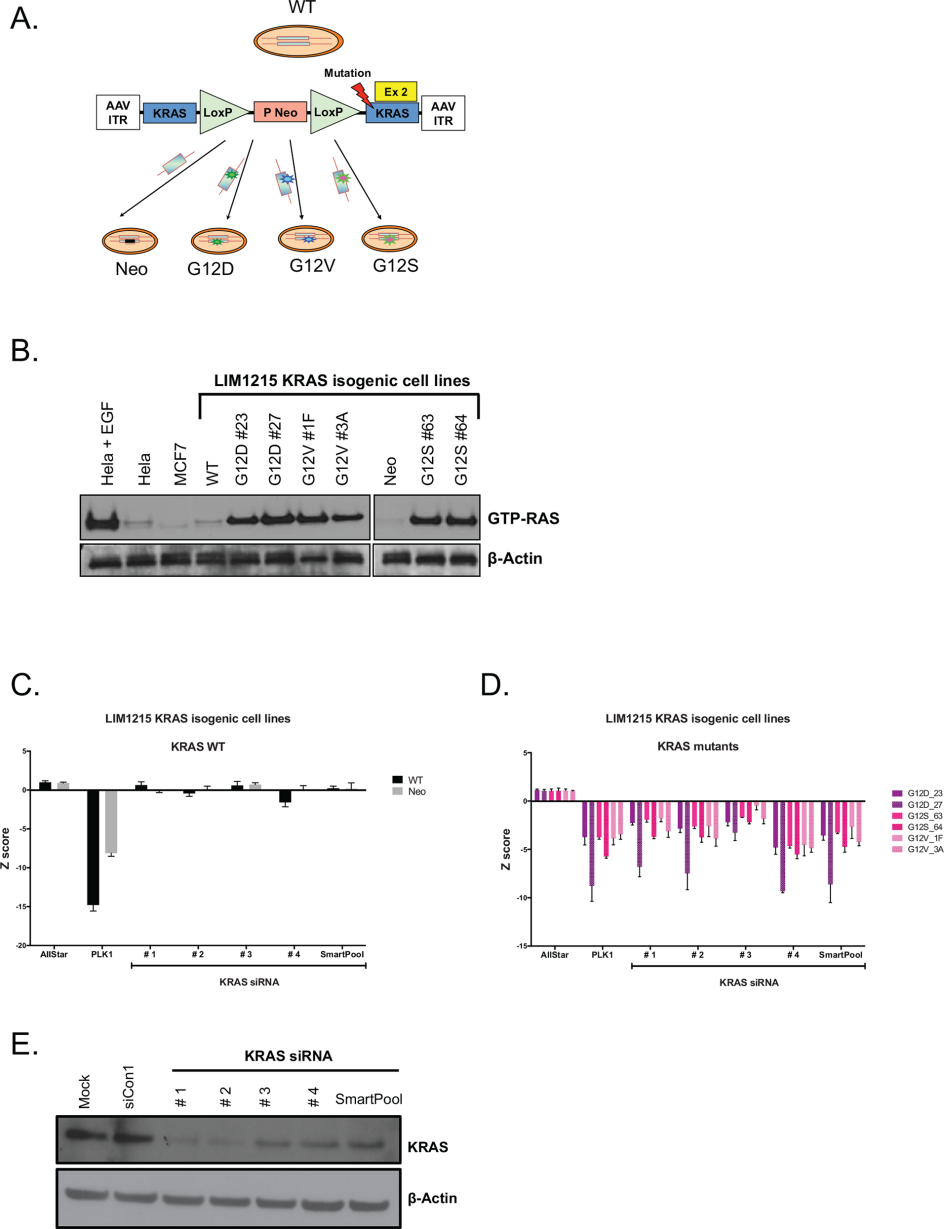
Characterization of LIM1215 isogenic cell lines. (A) Schematic of KRAS isogenic cell lines generation. *KRAS* mutations were introduced into the parental cell lines via r-AAV-mediated homologous recombination. A general structure of the targeting construct is represented. The resulting mutant *KRAS* allele is expressed from its endogenous promoter. The *Neo* cassette is removed from the genome of the targeted cells by Cre recombinase-mediated excision. AAV, adeno-associated virus; ITR, inverted terminal repeat; Neo, geneticin-resistance gene; P, SV40 promoter; triangles, loxP sites (Figure adapted from [27]). (B) RAS activation status of LIM1215 KRAS isogenic cell lines. Western blot showing active RAS (RAF1 GTP-bound) levels for LIM1215 KRAS isogenic cell lines. The RAF1 RAS binding domain (RBD) was used to precipitate GTP-RAS. The RAS activation status was tested for each clone with mutated KRAS. Precipitated RAS-GTP was detected by western blot using anti-RAS antibody. As a positive control, HeLa cells (RAS wild-type) were stimulated with epidermal growth factor (EGF) to activate the RAS pathway. HeLa and MCF7 cells (unstimulated) were used as negative controls. Total lysates were also immunoblotted with anti-β-Actin antibody as loading control. (C) and (D) KRAS dependence in the LIM1215 KRAS isogenic cell line models, obtained from the HT siRNA screen described in Fig 2. Bar graph of *KRAS* siRNA Z-score values across the LIM1215 *KRAS* WT and mutant isogenic cell lines, C and D respectively. KRAS dependence was greater in the cell lines carrying KRAS mutations than in WT cells. Error bars represent SEM from three independent experiments. (E) Western blot of KRAS in SW48 cells expressing *KRAS*-specific siRNAs. Multiple *KRAS* siRNA oligos and a pool efficiently suppressed KRAS expression showing that the siRNAs were on-target.

To identify candidate KRAS synthetic lethal interactions, we first generated and validated an isogenic cell line system to model the presence or absence of oncogenic *KRAS* mutations. We selected the KRAS wild type (WT) colorectal tumour cell line, LIM1215 as a model [26] and used AAV gene targeting to introduce one of three different oncogenic mutant *KRAS* alleles (enconding p.G12D, p.G12S and p.G12V) into the endogenous *KRAS* gene at position glycine 12 [27] (Fig 1A and S1 Fig). Replacement of one of the endogenous WT *KRAS* alleles with a mutant allele caused constitutive KRAS activation in all three cases, as assessed by measuring levels of active GTP-RAS (Fig 1B and S2A Fig). We also confirmed KRAS addiction in the *KRAS* mutant cell lines, by demonstrating cell growth inhibitory effects of siRNAs targeting KRAS (Fig 1C and 1D), after demonstrating that KRAS siRNA duplexes caused KRAS silencing (Fig 1E and S2B Fig).

**Fig 2:**
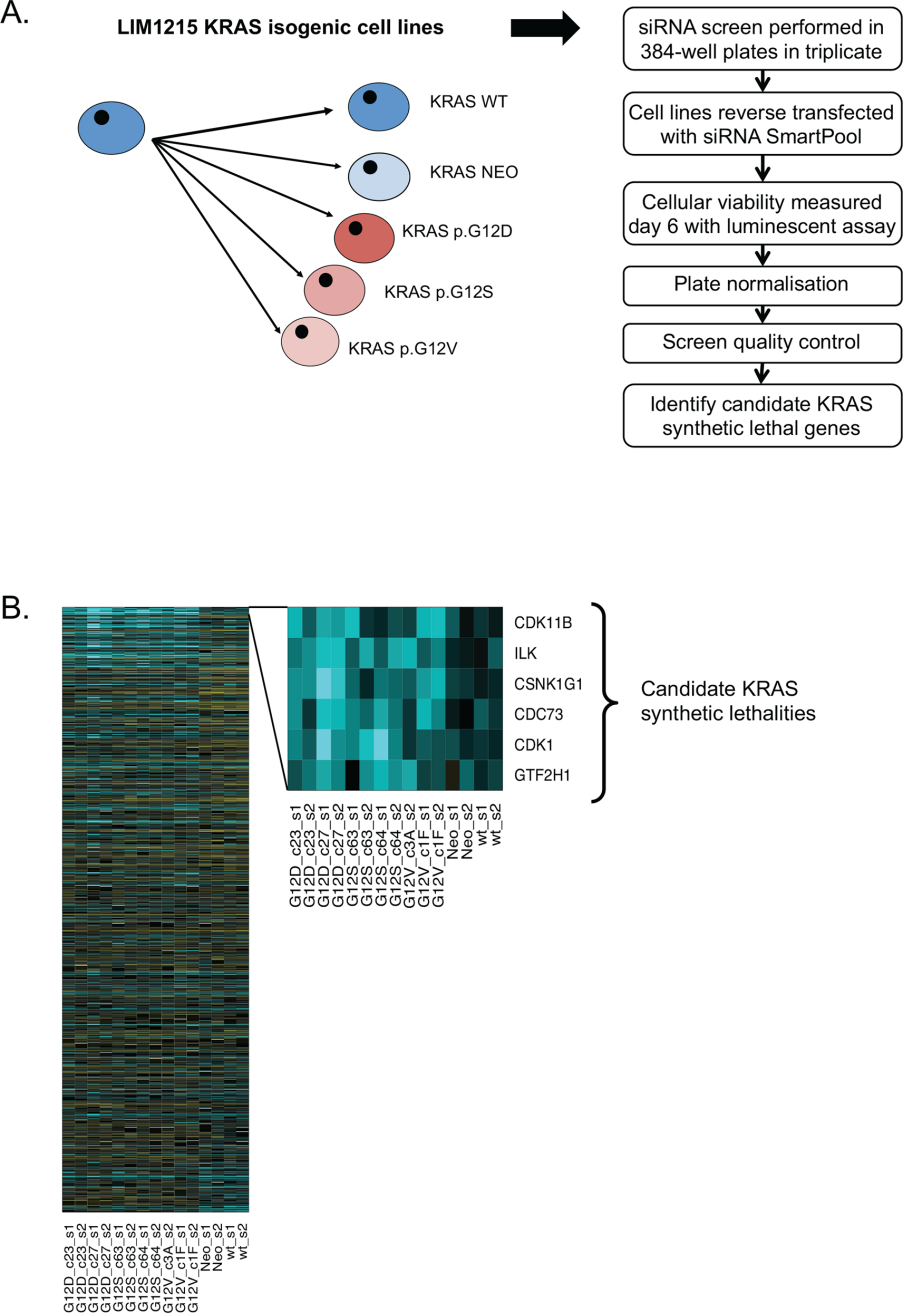
Identification of CDK1 as a KRAS synthetic lethality using functional genomic screens in the LIM1215 KRAS isogenic models. (A) Schematic describing the screen format. LIM1215 cells plated in 384 well plates were transfected with siRNA. Each transfection plate contained 300 experimental siRNAs supplemented with wells of non-targeting siCONTROL (siCON) and siRNA targeting PLK1 (positive control). Transfected cells were divided into three replica plates. Cell viability was assessed after six days using CellTiter-Glo Luminescent Cell Viability Assay (Promega). (B) Heatmap of siRNA z-scores for 15 screens used in the analysis. The rows of the heatmap correspond to siRNAs and are ordered by the difference in the KRAS mutant and non-mutant group median z-scores. The heatmap inset shows the six siRNAs selected for andditional analyses. These siRNAs were selected because they caused reduced viability in the KRAS mutant isogenic models (median z ≤ -2) but not in the WT and neo cell lines (median z ≥ -1).

We used parental LIM1215 KRAS WT (KRAS^WT/WT^) cells and of KRAS^WT/G12D^, KRAS^WT/G12S^ and KRAS^WT/G12V^ models in parallel siRNA screens to identify candidate synthetic lethal effects. For each genotype, we siRNA screened two independently derived clones. A LIM1215 clone encompassing a control targeting vector (LIM1215^neo^) was also screened in parallel. As a screening library, we used a 384 well plate arrayed siRNA library targeting 853 genes, predominantly protein kinase encoding genes. We focused on protein kinases given their inherent tractability as drug targets as well as their involvement in a wide range of intracellular signalling processes. Cells were reverse transfected with siRNA on day 0, and cell viability was estimated six days later using Cell Titre Glo reagent. In total we carried out two biological replicate screens for each clone, with each biological replicate encompassing three technical replica screens (Fig 2A). After assessing the quality of each screen by the calculation of Z’ values and Spearman rank correlation between replicates, we estimated the effect on cell inhibition of each siRNA in each clone and quantified these effects by calculating robust Z scores for each siRNA (Fig 2B and S3 Fig). The entire screening dataset is detailed in S1 Table.

To identify candidate KRAS synthetic lethal effects, we compared Z scores for each siRNA in *KRAS* mutant vs. WT clones. We used robust statistical thresholds to identify the most profound synthetic lethal effects, namely a median Z score across the p.G12 mutant cell lines (KRAS^WT/G12D^, KRAS^WT/G12S^ and KRAS^WT/G12V^) of ≤ -2 (approximately equal to a p < 0.05 effect) and a median Z score in the control, non-KRAS mutant lines of ≥ -1. Using these thresholds we identified six candidate KRAS SL genes; *Cyclin-Dependent Kinase 11B (CDK11B)*, *Integrin-Linked Kinase (ILK)*, *Casein Kinase 1*, *Gamma 1 (CSNK1G1)*, *Cell Division Cycle 73 (CDC73)*, *Cyclin Dependent Kinase 1 (CDK1)* and *General Transcription Factor IIH*, *Polypeptide 1*, *62kDa (GTF2H1)* (Table 1 and Fig 2B).

**Table 1:** Candidate *KRAS* synthetic lethal genes identified in LIM1215 KRAS isogenic cell lines siRNA screens. The table shows the median Z scores for the KRAS WT and mutant cells and the respective Delta median Z scores.

Gene Symbol	Mutant median Z scores	WT median Z scores	Delta median Z scores
CDK11B	-2.59	-0.74	-1.85
ILK	-2.39	-0.61	-1.79
CSNK1G1	-2.31	-0.79	-1.53
CDC73	-2.09	-0.64	-1.46
CDK1	-2.11	-0.80	-1.32
GTF2H1	-2.05	-0.86	-1.19

### Identification of robust KRAS synthetic lethality effects

**Fig 3:**
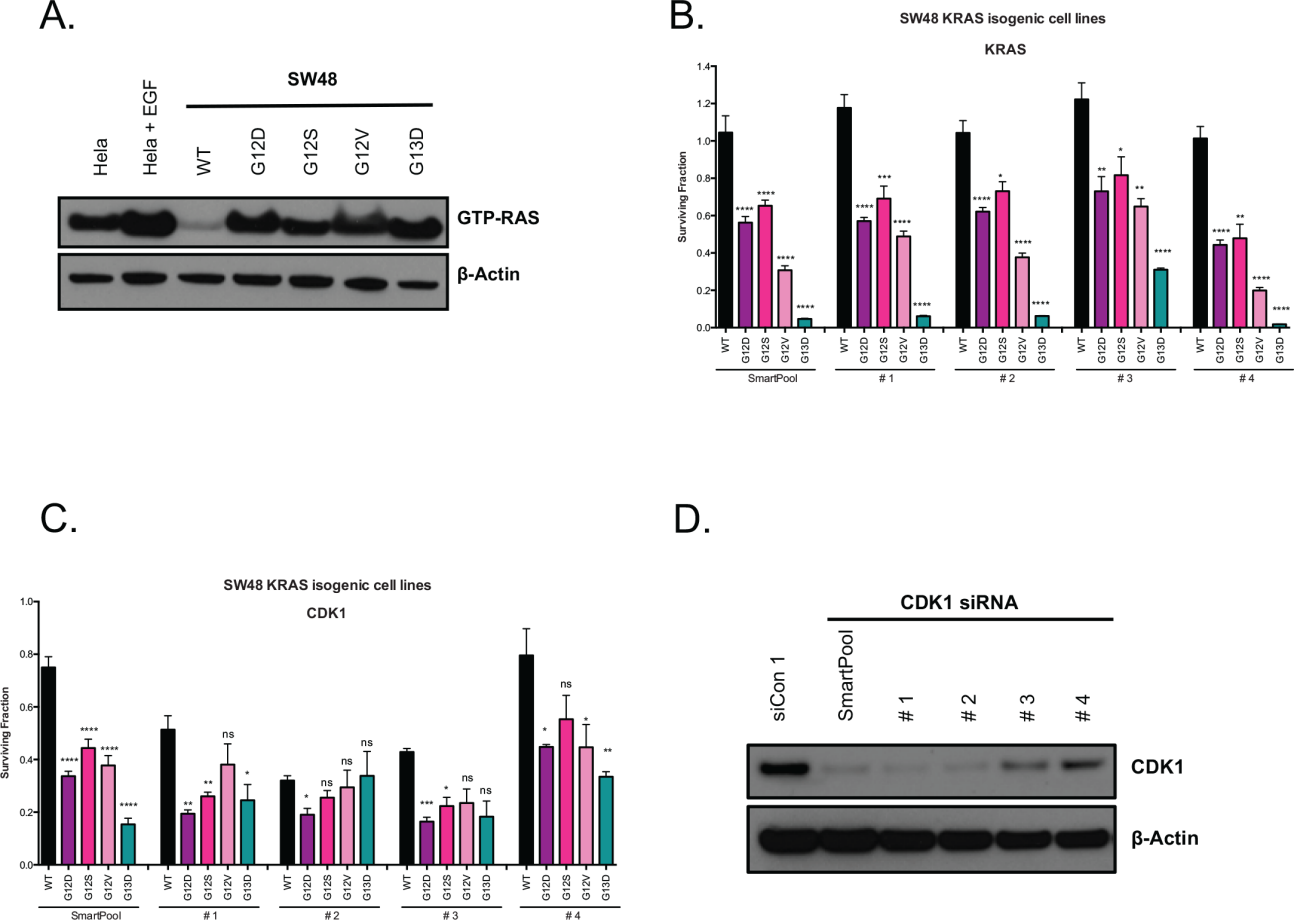
Validation of the CDK1 hit from the LIM1215 siRNA screen in SW48 isogenic cell lines. (A) GTP-RAS assay showing the RAS activation status of SW48 KRAS isogenic cell lines. (B) KRAS dependence in the SW48 KRAS isogenic cell lines (*P<0.05, **P<0.01, ***P<0.001, ****P<0.0001, Student’s t-test for comparison between each *KRAS* mutant and the WT cell lines). (C) CDK1-specific siRNAs suppress CDK1 expression. Cell viability after CDK1 depletion in SW48 isogenic KRAS cell lines (ns, not statistically significant, *P<0.05, **P<0.01, ***P<0.001, ****P<0.0001, Student’s t-test for comparison between each *KRAS* mutant and the WT cell lines). Error bars represent SEM from three independent experiments. (D) Western blot of CDK1 in SW48 parental cells expressing CDK1-specific siRNAs.

One of the challenges in the identification and validation of synthetic lethal targets in cancer is distinguishing “hard” synthetic lethal effects that are relatively resistant to other genetic or epigenetic changes, from “soft” synthetic lethal effects that are readily abrogated by such changes [23]. Our intention was to identify relatively robust, hard synthetic lethal effects. We therefore assessed each of the six candidate KRAS SLs identified in the LIM1215 screen in a second KRAS isogenic system consisting of a second colorectal tumour cell model, SW48, in which KRAS mutant alleles were also introduced by AAV gene targeting. From a parental SW48 (KRAS^WT/WT^) clone, we generated isogenic KRAS^WT/G12D^, KRAS^WT/G12S^, KRAS^WT/G12V^ and KRAS^WT/G13D^ clones and assessed RAS activation in these clones (Fig 3A and S4A Fig) as well as KRAS addiction (Fig 3B), as before.

Using this second isogenic system, we assessed the effect of six candidate KRAS SLs using siRNA gene silencing. From these six candidates only Cyclin-Dependent Kinase 1 (CDK1) validated, as the CDK1 siRNA gave a more profound effect in the SW48 KRAS mutant cells than WT cells (Fig 3C). We did however note that CDK1 siRNA, whilst having a greater effect in KRAS mutant cells, did also elicit some level of cell inhibition in SW48 KRAS WT cells, and therefore CDK1 may be regarded as synthetic sick with KRAS, rather than synthetic lethal. CDK1, also known as CDC2, is a cyclin dependent kinase (CDK) implicated in G_2_/M transition and progression through mitosis. CDK1 is activated by A-type cyclins in late G_2_, an event that drives mitosis [28, 29]. While CDK1 has been primarily implicated in the G_2_/M transition, a number of studies have demonstrated that CDK1 is also involved in G_1_/S transition [30–32]. CDK1 binds interphase (D-type and E-type) cyclins, and these interactions are increased in cells deficient in interphase related CDKs, such as CDK2 or CDK4. Therefore, CDK1 may be crucial for the regulation of growth-inducing transcription as well as DNA replication and repair [30–33].

**Fig 4:**
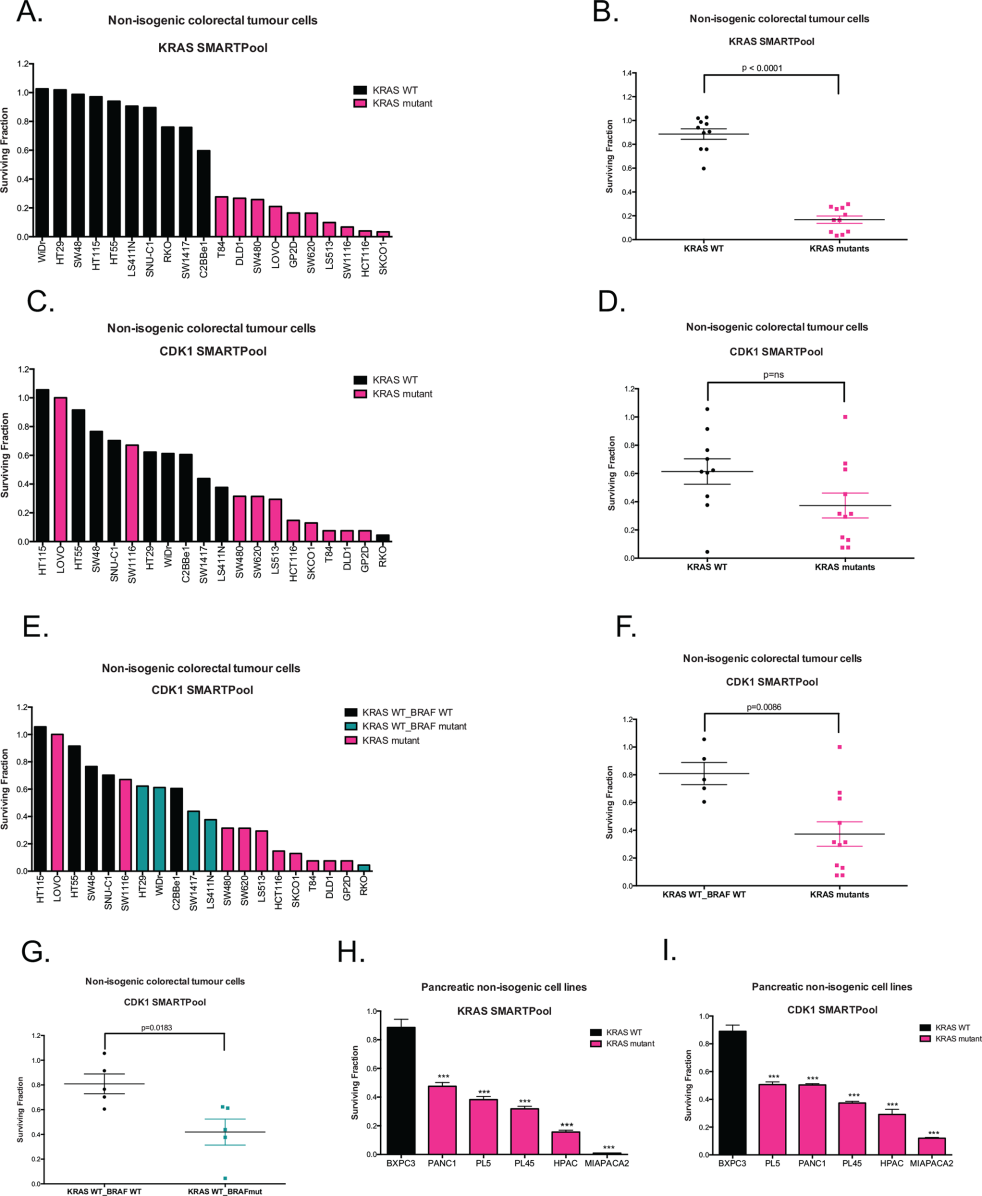
Effects of CDK1 depletion in non-isogenic KRAS mutant cell models. (A) and (B) Waterfall and scatter plots, respectively, of a panel of non-isogenic colorectal cell lines showing a statistically significant difference in surviving fraction in response to KRAS depletion by siRNA between the *KRAS* WT (black) and mutant (pink) cell lines (P<0.0001, Student’s t-test for comparison between *KRAS* mutant and WT cell lines). (C) and (D) Waterfall and scatter plots, respectively, showing the surviving fractions of a panel of non-isogenic CRC cell lines after CDK1 depletion by siRNA. Black bars represent *KRAS* WT cell lines and pink bars represent *KRAS* mutant cell lines (ns, not statistically significant, Student’s t-test for comparison between *KRAS* mutant and WT cell lines). (E) Waterfall plot of the same CRC cell line panel as described in 4C, but with the *KRAS* WT cells divided into *KRAS* WT/*BRAF* WT (black), *KRAS* WT/*BRAF* mutant (green). (F) Scatter plot of the panel of CRC non-isogenic cell lines, without the *KRAS* WT/*BRAF* mutant cells, showing that the difference between the *KRAS* mutant (pink) and WT (black) cells to CDK1 depletion by siRNA is statistically significant (**P>0.01, Student’s t-test for comparison between *KRAS* mutant and WT cell lines.). (G) Scatter plot demonstrating a statistically significant difference in survival between *KRAS* WT/*BRAF* WT (black) and *KRAS* WT/*BRAF* mutants (green) after CDK1 depletion (*P>0.05, Student’s t-test for comparison between *BRAF* mutant and WT cell lines.). (H) Waterfall plot of pancreatic cell lines showing that the *KRAS* mutant cells (pink) were significantly more sensitive to KRAS depletion by siRNA than the *KRAS* WT cells (black) (***P<0.001, Student’s t-test for comparison between each *KRAS* mutant cell lines and the WT cell line). (I) Waterfall plot of pancreatic cell lines showing that the *KRAS* mutant cells (pink) were significantly more sensitive to CDK1 depletion by siRNA than the *KRAS* WT cells (black) (***P<0.001, Student’s t-test for comparison between each *KRAS* mutant cell lines and the WT cell line). Surviving fractions normalized to siControl1 transfected cells. Error bars represent SEM from three independent experiments.

Having established that multiple independent CDK1 siRNA duplexes caused CDK1 silencing and KRAS selectivity (Fig 3C and 3D and S4B Fig), we assessed cell growth inhibition in a panel of 20 genetically diverse colorectal tumour cell line models transfected with multiple different CDK1 siRNAs (S2 Table). To confirm KRAS addiction in each cell line, we determined the cell growth inhibitory effects of KRAS depletion (Fig 4A and 4B). Next, we compared the growth inhibitory effects of CDK1 siRNA in the *KRAS* mutant (n = 10) and WT cohorts (n = 10). We found that although CDK1 siRNA did inhibit multiple *KRAS* mutant models it also had inhibitory effects in *KRAS* WT models, HT29, WiDr, LS411N, SW1417 and RKO (Fig 4C and 4D), an observation inconsistent with the KRAS SL effects observed in the isogenic systems (Fig 3C). However, a more in-depth analysis of this data indicated that those KRAS WT models that were sensitive to CDK1 siRNA had oncogenic p.V600E *BRAF* mutations (Fig 4E). BRAF is a KRAS effector and part of the mitogen-activated protein kinases (MAPK) RAS-RAF-MEK-ERK signalling cascade. Upon KRAS activation, BRAF is recruited to the cell membrane, where its phosphorylation status is altered, an event that subsequently causes MEK1/2 and ERK activation [3].

Reanalysis of the colorectal cell line dataset suggested that CDK1 silencing was indeed selective for the *KRAS* mutant/ *BRAF* WT cohort compared to the *KRAS* WT/*BRAF* WT cohort (p<0.01, Student’s t-test, Fig 4F) and that CDK1 siRNA was also selective for the *KRAS* WT/*BRAF* mutant models compared to *KRAS* WT/*BRAF* WT models (p<0.05, Student’s t-test, Fig 4G). It seems possible that both KRAS and BRAF drive a shared process or series of effects that induce CDK1 synthetic lethality. We also noted that there was some variation in the inhibitory effect of CDK1 siRNA across the panel of *KRAS* mutant/ *BRAF* WT cohort. This might suggest that in addition to KRAS being a determinant of CDK1 siRNA sensitivity, additional determinants of sensitivity might also enhance or decrease the extent of synthetic lethality.

We also assessed the KRAS/CDK1 SL in cell models of pancreatic ductal adenocarcinoma (PDAC), a disease where where somatic *KRAS* mutations are prevalent in approximately 90% of cases [34]. We found that in a cohort of six PDAC cell line models, the five KRAS mutant models (S2 Table) were not only more sensitive to *KRAS* siRNA but also more sensitive to *CDK1* siRNA than the *KRAS* WT BxPC3 PDAC model (Fig 4H and 4I).

### Chemical inhibition of CDK1 causes KRAS synthetic lethality *in vitro* and *in vivo*

**Fig 5:**
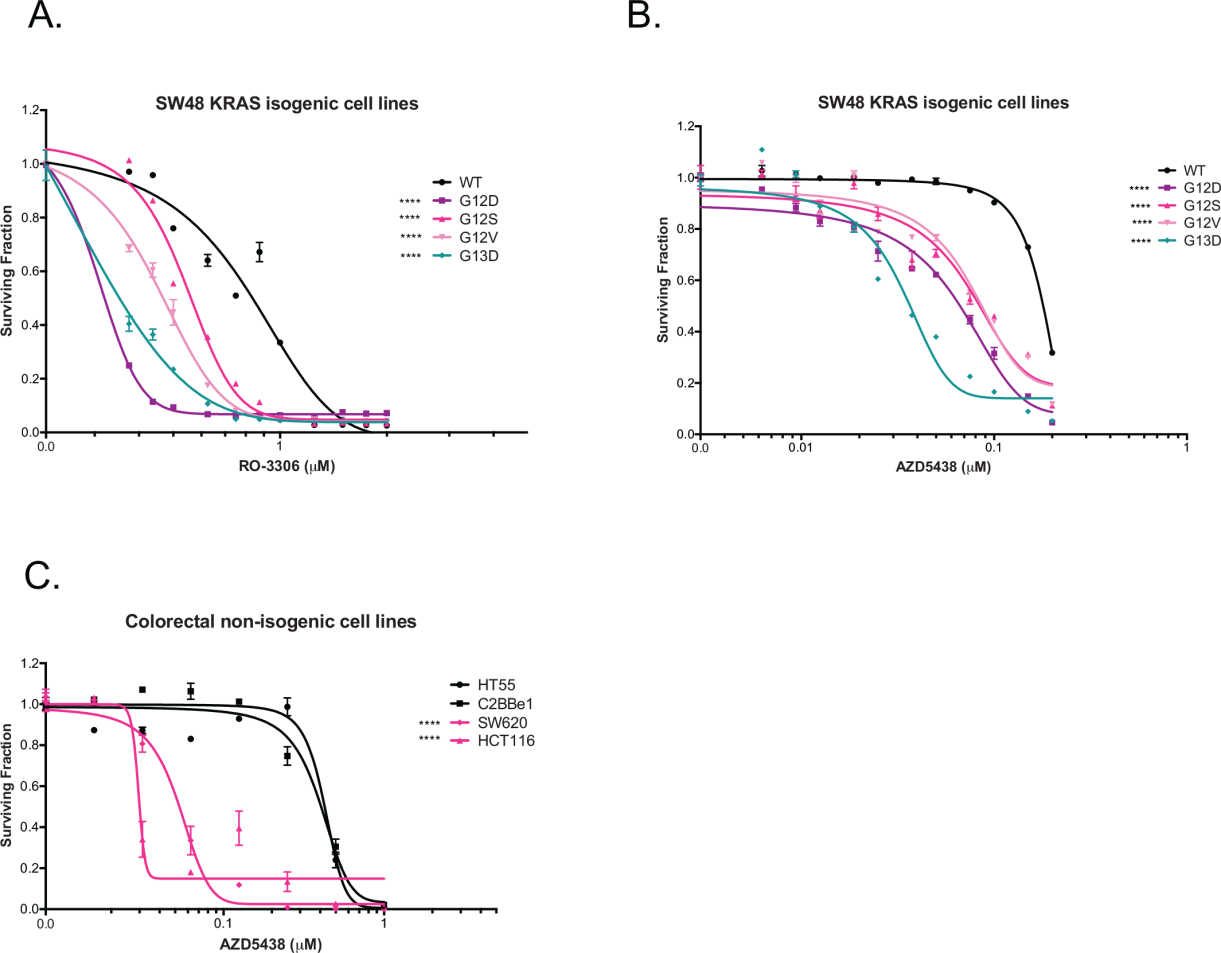
CDK inhibitors sensitivity profile in SW48 KRAS isogenic cell lines. (A) Exposure of SW48 isogenic cell lines to RO-3306 in a fifteen-day colony formation assay. (B) Exposure of SW48 isogenic cell lines to inhibitor AZD5438, in a fifteen-day colony formation assay. (C) Drug-dose response curves of CRC cells after AZD5438 exposure in a fifteen-day colony formation assay. ****P<0.0001, Two-way ANOVA. Error bars represent SEM of three technical replicates. All the experiments were performed two independent times with three technical replicates.

To assess the therapeutic potential of these observations, we assessed the sensitivity of KRAS mutant models to small molecule inhibitors of CDK1. Here we used two chemically distinct inhibitors, RO-3306, a CDK1 inhibitor [35] and AZD5438, a CDK1/2 and 9 inhibitor [36]. We found both inhibitors were KRAS selective in the SW48 isogenic system (Fig 5A and 5B, S3 Table), validating the siRNA results. Further assessment of CDK1 inhibitor sensitivity in panels of non-isogenic CRC and PDAC cell lines showed a KRAS mutant selective response after AZD5438 exposure. For example, the CRC mutant cell lines, SW620 and HCT116, showed a significant difference in sensitivity when compared to the KRAS WT cells, HT55, C2BBe1, P<0.0001, two-way ANOVA (Fig 5C, S3 Table). The PDAC KRAS mutant cell lines, HPAC, PL5 and PL45, also showed a significant difference in sensitivity when compared to the KRAS WT cells, BxPC3, P<0.001, P<0.0001 and P<0.01, respectively, two-way ANOVA (S5A Fig, S3 Table). Also we found that the *KRAS* WT/*BRAF* mutant models RKO and LS411N showed sensitivity to as well as sensitising to CDK1 siRNA results previously discussed (S5B Fig). These results suggest that colorectal *BRAF* mutant cells, together with the *KRAS* mutant cell lines, are sensitive to CDK1 inhibition.

### KRAS mutant tumour cells exhibit elevated CDK1 activity and exhibit an S phase entry defect upon CDK1 inhibition

**Fig 6:**
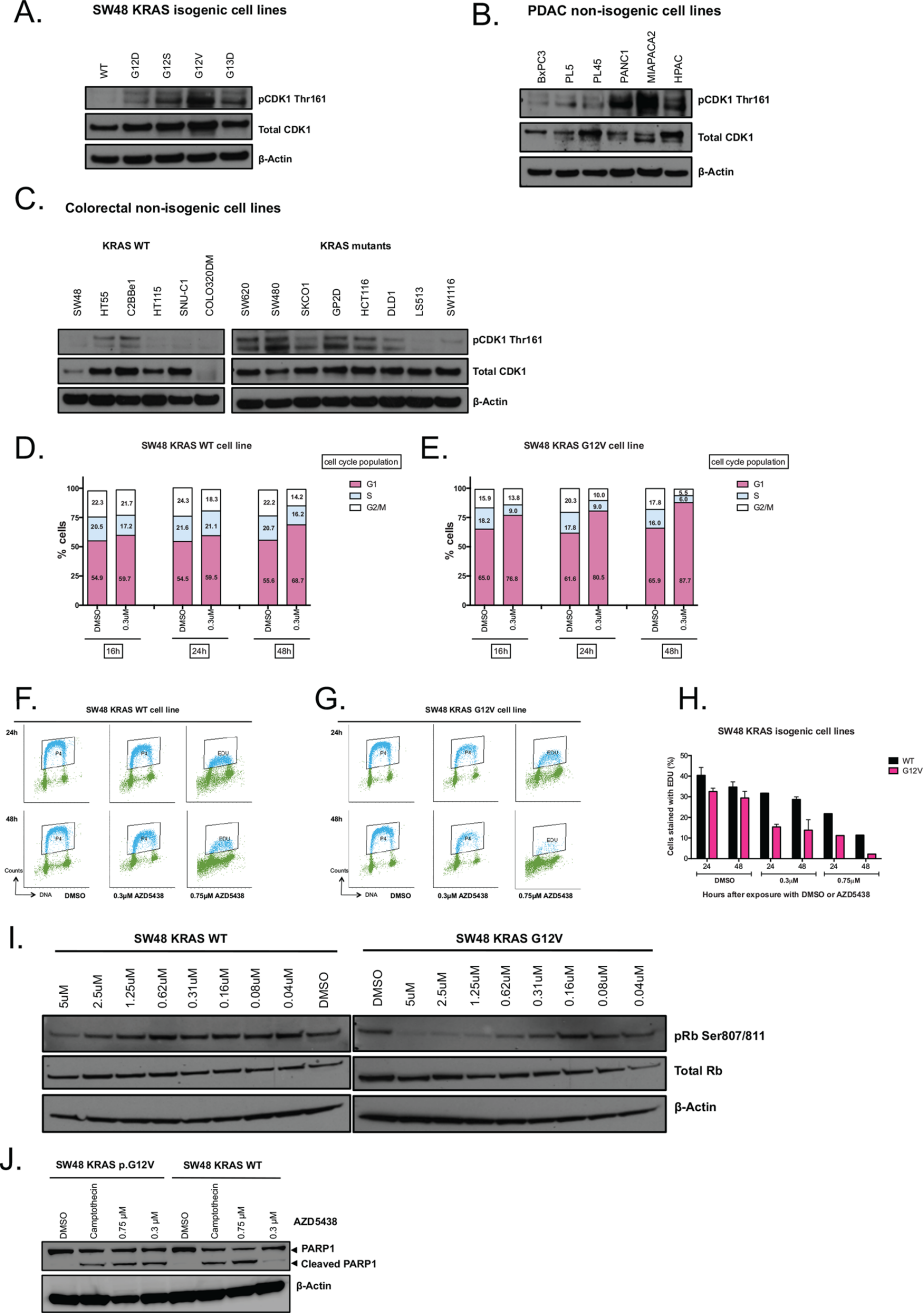
(A—C). CDK1 phosphorylation levels in KRAS mutant and WT cells as shown by Western blot analysis of total cell protein lysates from SW48 KRAS isogenic (A), non-isogenic pancreatic tumour cell lines (B) and non-isogenic colorectal cell lines (C). Western blots were probed for CDK1 (pThr161 CDK1 and total CDK1). β-actin detection was used as a loading control. (D and E) Bar graphs illustrating the percentage of cells in G1, S and G2/M cell cycle phases in SW48 KRAS WT or p.G12V mutant cell lines after AZD5438 exposure. SW48 KRAS WT (D) and p.G12V (E) were exposed to 0.3 μM AZD5438 or DMSO for 16, 24 and 48 hours after which cell cycle profiles were assessed by propidium iodide (PI) staining and flow cytometry. The KRAS p.G12V mutant cells showed a decrease in S and G2-fractions after exposure to AZD5438 when compared to control (DMSO) treated cells and to KRAS WT cells (AZD5438 and DMSO). (F—H) DNA synthesis in SW48 KRAS WT and p.G12V cell lines after AZD5438 exposure. (F) and (G) 5-ethynyl-2'-deoxyuridine (EDU)/ PI FACS plots in SW48 KRAS WT (F) and p.G12V mutant cells exposed to AZD5438 0.3 μM and 0.75 μM, or DMSO for 24 and 48 hours. After AZD5438 exposure, EDU/PI profiles were assessed by flow cytometry. EDU stained cells are represented in blue. (H) Bar graph illustrating the percentage of cells stained with EDU over time for both SW48 KRAS WT and p.G12V mutant cells. (I) Western blot illustrating the phosphorylation of Retinoblastoma protein (pRb) in SW48 KRAS WT and p.G12V mutant cell lines after AZD5438 exposure. Cells were exposed to AZD5438 for two hours after which total cell lysates were generated and western blotted as shown. Detection of β-Actin was used as a loading control. The levels of Rb phosphorylation on Ser807/811 were decreased in the KRAS p.G12V cells when compared to the WT cells, after AZD5438 2 hours exposure. (J) Western blot illustrating PARP1 cleavage in SW48 KRAS WT and p.G12V mutant cells after 72h of AZD5438 exposure. Cells were exposed to AZD5438 for two hours after which total cell lysates were generated and western blotted as shown. Exposure to camptothecin was used as a positive control.

To investigate the molecular basis of the KRAS/CDK1 SL, we assessed the extent of CDK1 protein expression and phosphorylation using western blot analysis. We found the extent of CDK1 phosphorylation at Thr161 to be enhanced in *KRAS* mutant cells compared to WT cells in the SW48 KRAS isogenic model, PDAC and CRC non-isogenic tumour cell lines (Fig 6A–6C and S6 and S7 Figs). CDK1 phosphorylation at Thr161 is caused by the activity of CDK-activating kinase (CAK), which leads to activation of CDK1 and can improve its binding to cyclins [37]. We therefore concluded that there was an enhanced level of CDK1 activity in KRAS mutant cells.

CDK1 regulates several steps of cell cycle progression [32]. Variations in levels of activated CDK1 could conceivably cause differences in cell cycle progression and timing. We decided to examine the cell cycle profile of SW48 *KRAS* p.G12V mutant and WT cells either in the presence or absence of CDK1 inhibitor. In the absence of CDK1 inhibition *KRAS* p.G12V mutant cells showed a modest increase in the proportion of cells in G_1_ when compared to WT cells (after 48h, 65.9% versus 55.6%, respectively). This difference was enhanced when cells were exposed to AZD5438 (G_1_ fraction in p.G12V KRAS mutant cells exposed to 0.3 μM = 87.7% compared to 68.7% for similarly treated WT cells). Concomitantly, we noticed a significant reduction in the proportions of *KRAS* p.G12V mutant cells in the S- and G_2_/M phases when compared to WT cells after cells were exposed to AZD5438 (S fraction in p.G12V KRAS mutant cells exposed to 0.3 μM = 6% compared to 16.2% for similarly treated WT cells, p = 0.034, Student’s t-test) (Fig 6D and 6E, S8 Fig).

As the proportion of cells in S-phase after exposure to AZD5438 was lower in the *KRAS* p.G12V cells than in the WT cells, we examined S phase progression by measuring DNA synthesis using the Click-iT Plus 5-ethynyl-2'-deoxyuridine (EDU) assay. In this assay, the fluorescently labelled thymidine analogue EDU is incorporated into newly synthesized DNA, an event that can be monitored by measuring fluorescence [38]. DNA content analysis together with EDU analysis suggested that AZD5438 caused a significant defect in S-phase progression in *KRAS* mutant cells when compared to both untreated mutant and WT cells (Fig 6F–6H). These results suggested that the inhibition of CDK1 by AZD5438 caused a disruption in DNA synthesis in the *KRAS* mutant cells, an effect that was not as profound in *KRAS* WT cells.

So that cells can proceed into S-phase, Retinoblastoma protein (Rb) is phosphorylated by either CDK1 or CDK2 at Ser807 and Ser811. This post translational modification inactivates Rb and facilitates cell cycle progression [39]. We therefore determined whether the enhanced G_1_ fraction and reduction in S-phase cells caused by AZD5438 exposure in KRAS mutant cells was also associated with differences in Rb phosphorylation (Fig 6I). In *KRAS* p.G12V mutant cells, Rb Ser807 and Ser811 phosphorylation were both reduced by AZD5438 exposure. In KRAS WT cells, Rb phosphorylation was maintained in the face of AZD5438 exposure (Fig 6I and S9A Fig). Taken together, these findings suggested that *KRAS* p.G12V isogenic cells have a delay in entering S-phase and become arrested in G_1_-phase upon exposure to AZD5438.

We further assessed the mechanism of cell inhibition caused by AZD5438. We found that a high, non KRAS selective concentration of AZD5438 caused PARP cleavage in both KRAS WT and mutant cells, but a KRAS selective concentration of AZD5438 caused apoptosis in the KRAS mutant cell line but not in the KRAS model (Fig 6J and S9B Fig). This suggested that the KRAS selective effect of AZD5438 might be caused by G_1_ arrest followed by apoptosis.

### Synthetic lethality of other CDK inhibitors with KRAS

The druggable nature of CDKs, along with the recurrent dysregulation of CDK activity in human cancer, has led to intensive efforts to develop selective CDK inhibitors [40]. CDK4 has already been proposed as being synthetic lethal with KRAS [17] and here we identified CDK1 as a KRAS SL partner, reinforcing the potential of cyclin dependent kinases as KRAS synthetic lethal targets.

**Fig 7:**
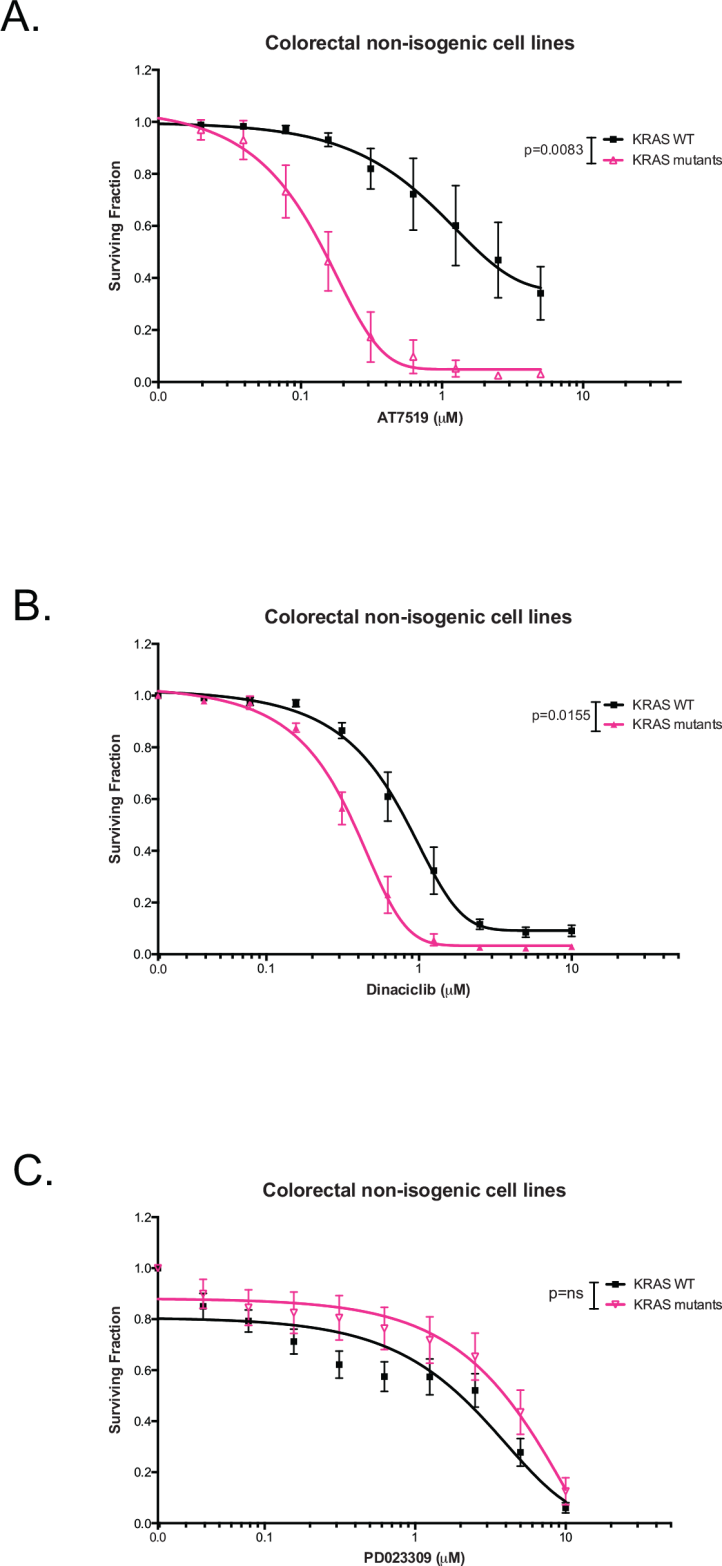
Response to three second-generation CDK inhibitors in CRC cell lines. (A) AT7519, (B) dinaciclib and (C) PD023309 median of drug-dose response curves of *KRAS* WT and mutant cells from a five-day cell viability assay to assess the KRAS selectivity of the CDK inhibitors in ten colorectal cell lines, four KRAS WT (black) and six mutant (pink) cell lines. (ns not-significant, Two-way ANOVA) Experimental conditions were repeated in triplicate. Error bars represent SEM. Only AT7519 and dinaciclib showed KRAS selectivity in the CRC cell lines.

In order to identify whether other small molecule CDK inhibitors elicited the same KRAS selective effects as AZD5438, we assessed the tumour cell inhibitory effects of a range of CDK inhibitors: (i) AT7519, a CDK1/2/4/5/9 inhibitor [41]; (ii) dinaciclib, a CDK1/2/5/9 inhibitor [42]; and as a control (iii) PD023309, a CDK4/6 inhibitor [43], in a panel of CRC non-isogenic tumour cell lines (Fig 7 and S10 Fig). AT7519, a CDK1/2/4/5/9 inhibitor [41], showed the greatest selectivity for KRAS mutant tumour cells. The average KRAS mutant selectivity of AT7519 was 6.5-fold compared to WT cells (p = 0.0083, two-way ANOVA) (Fig 7A, S10A Fig, S4 Table). Dinaciclib, a CDK1/2/5/9 inhibitor, also showed KRAS selectivity although this was less profound than for AT7519 (p = 0.0155, two-way ANOVA) (Fig 7B, S10B Fig, S4 Table). Finally, PD023309, a CDK4/6 inhibitor, did not show KRAS selectivity in the CRC tumour cell lines tested (p = ns, two-way ANOVA) (Fig 7C, S10C Fig, S4 Table), although Barbacid and colleagues have previously demonstrated that PD023309 inhibits the proliferation of KRAS p.G12V-induced non-small cell lung cancer (NSCLC) cells [17].

### CDK inhibition reduces tumour growth in KRAS mutant xenografts

**Fig 8:**
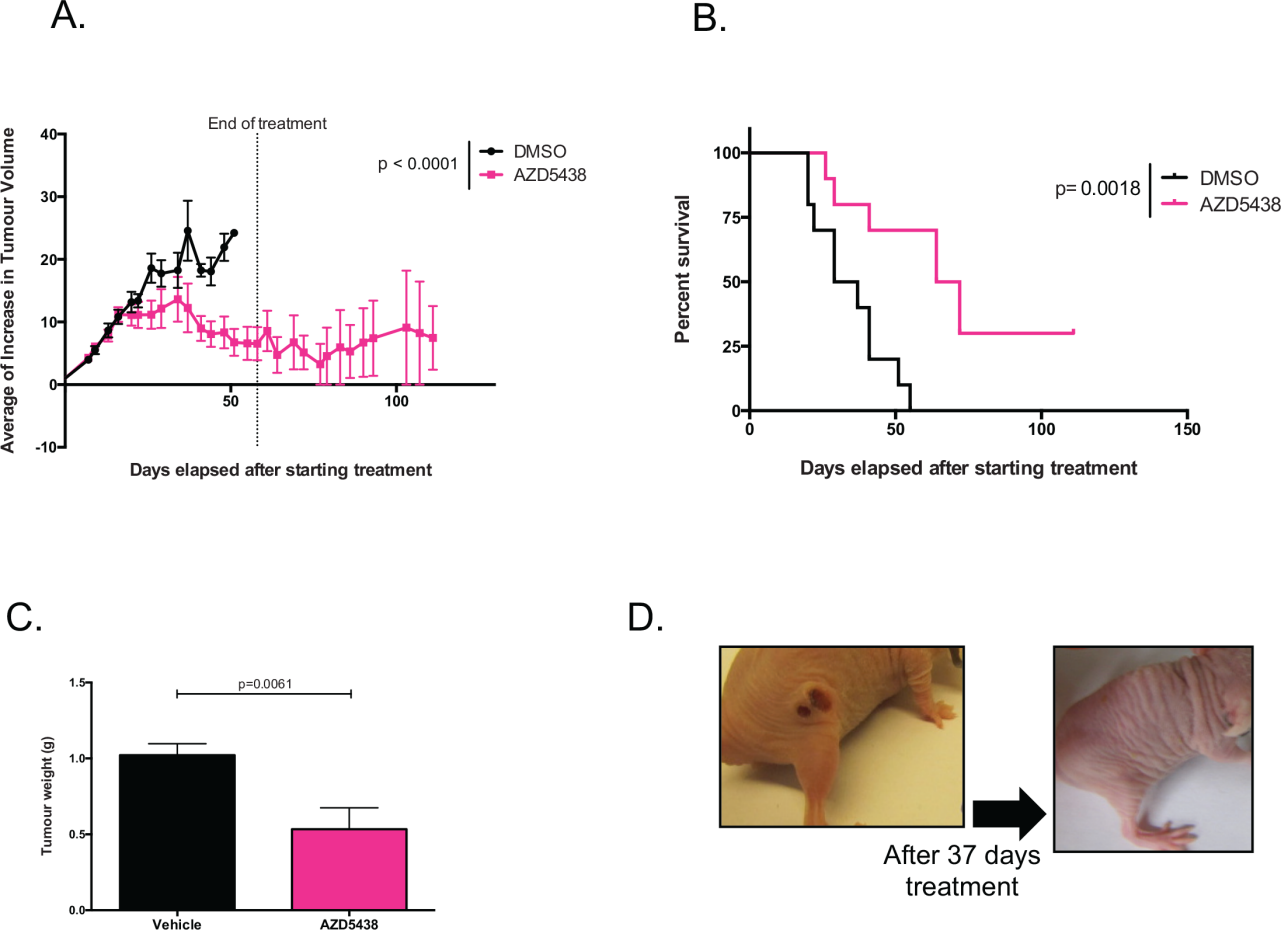
*In vivo* efficacy of AZD5438 in SW620 cell xenografts. KRAS mutant xenografts were treated with vehicle (DMSO in black) or AZD5438 (20mg/kg/day (in pink)). (A) Mean of increase in tumour volume relative to initial tumour volume. A one-way ANOVA was performed to compare both arms and the difference in the SW620 xenografts was statistically significant (p = 0.017). (B) Survival curves showing a statistically significant difference between the treated and vehicle arms, where the mice in the drug arm had an increase in overall survival using a Log-rank Mantel-Cox test (p = 0.0018 in SW620 xenografts). (C) Average final tumour weight. There is a significant difference between the vehicle and treatment arms (**p < 0.01, t-test). Error bars represent SEM. (D) Photograph of a mouse treated with AZD5438, where the tumour disappeared completely, after 37 days of treatment.

On the basis of the *in vitro* selective effects of AZD5438 on KRAS mutant cells, we assessed *in vivo* efficacy of AZD5438. We xenografted SW620 (*KRAS* p.G12V) colorectal tumour cells into immunocompromised mice and once tumours had established, we treated animals with AZD5438. Specifically, animals with established SW620 tumours (80 mm^3^) were randomized into one of two cohorts and treated with either 20 mg/kg/day AZD5438 or vehicle (n = 10 in each cohort) (Fig 8). The growth of SW620 xenografts, was clearly inhibited by AZD5438 treatment with some mice exhibiting complete tumour eradication (Fig 8A). SW620 xenografted mice, treated with AZD5438 also showed a survival advantage compared to vehicle treated mice (Fig 8B). Moreover, the weight of tumours in the AZD5438 treated mice cohort was in general 60% less than in vehicle treated mice (p < 0.01, t-test) (Fig 8C), suggesting that AZD5438 could inhibit a KRAS mutant tumour *in vivo*. To confirm that this *in vivo* efficacy effect might be KRAS selective, we assessed the ability of AZD5438 to inhibit established xenografts from either KRAS WT or p.G12V mutant SW48 cells (S11 Fig). Whilst AZD5438 had no effect on KRAS WT xenografts, we observed a statistically significant inhibition of KRAS mutant xenografts compared to vehicle treatment (p = 0.0023, one-way ANOVA).

## Discussion

Here, we describe a series of experiments aimed at identifying novel dependencies in KRAS mutant tumour cells. Using genetic screens in LIM1215 KRAS isogenic cell lines we identified novel KRAS synthetic lethal effects, including CDK1. One weakness of the HT screen might be the use of engineered models of KRAS mutation, rather than the use of models with naturally occurring KRAS mutations. It is possible that these engineered models do not fully replicate all of the KRAS synthetic lethal effects found in real human tumours. However, we do note that we also observed the KRAS selective effect of CDK1 inhibition in tumour cell lines with naturally occurring KRAS mutations (Fig 4, Fig 5 and Fig 7). CDK1 was validated using a different KRAS isogenic cell model (SW48) as well as a series of non-isogenic CRC and PDAC tumour cell models. This analysis suggested that the CDK1/KRAS synthetic lethal effect was not restricted to isogenic systems but also operated in a variety of different, genetically diverse, KRAS mutant tumour cells. One priority in identifying SL effects is to discriminate SLs that are easily abrogated by additional genetic and epigenetic alterations (soft SLs) from those that are somewhat more resistant to these changes (hard SLs) [23]. The analysis in the non-isogenic tumour cell panel suggested that the CDK1/KRAS SL was a relatively hard synthetic lethal effect. CDK1 forms active complexes with A-, B-, E-, and D-type cyclins [30], as part of its critical role in cell cycle progression. Inhibition of CDK1 with AZD5438, led to a marked reduction in the proportion of cells in S and G_2_/M phases of the cell cycle in KRAS mutant cells. This was accompanied by an increased in the proportion of cells in G_1_. Moreover, western blotting revealed that AZD5438 decreased Rb phosphorylation levels in KRAS mutant cells compared to KRAS WT cells. As cells require hyperphosphorylation of Rb to pass the restriction point in G_1_ prior to entering S-phase, the reduction in Rb phosphorylation could provide an explanation as to the cell cycle arrest, which could be a possible cause for the reduced proportion of KRAS mutant cells in S-phase [39]. We determined the mechanism of cell inhibition to be apoptosis, where western blotting showed increased PARP cleavage in the SW48 p.G12V cells. We also assessed the anti-tumour effect of AZD5438 on human tumour xenografts to access the KRAS selectivity of this compound in an *in vivo* setting. These experiments were performed in immunocompromised mice with SW620 cell together with SW48 KRAS isogenic p.G12V and WT cells xenografts, and have confirmed the KRAS selective inhibitory effect of AZD5438, supporting the *in vitro* results. Overall the results from this experiment suggested disease stabilisation after AZD5438 treatment, but in some cases complete tumour inhibition was observed. Already a number of other synthetic lethal interactions involving cyclin dependent kinases have been proposed. Perhaps the most notable synthetic lethal interaction involving CDK1 described to date is between CDK1 and the oncogenic transcription factor, MYC [44]. In this particular case, MYC overexpressing tumour cells appear to be reliant upon the activity of the inhibitor of apoptosis protein (IAP), BIRC5 (survivin) a CDK1 target [44]. This particular synthetic lethality can be elicited in triple negative breast tumour cells using the clinical CDK1,2,5,9 inhibitor dinaciclib [45]. As well as providing a potential route to targeting MYC driven tumours, CDK1 inhibition has also been proposed as a route to causing chemosensitivity, enhancing the effects of PARP inhibitors [46] as well as PI3-kinase inhibitors [47]. Here we show a KRAS/CDK1 synthetic lethality that can be elicited with small molecule CDK1 inhibitors might also be added to the list of potential utilities for small molecule CDK inhibitors in cancer.

## Supporting Information

S1 Fig Sequence for *KRAS* mutations in LIM1215 KRAS isogenic cell lines. The introduced genetic alterations in the targeted cells was determined by RT-PCR and sequencing of the *KRAS* transcript. The different introduced mutations are indicated for each cell line with a black arrow in the chromatograms.
(PDF)

S2 Fig Uncropped western blots from the main figures. (A) Fig 1B. (B) Fig 1E.
(PDF)

S3 Fig siRNA screen analysis flowchart. Cell viability was assessed at day six by CellTiter-Glo (Promega) luminescence reading. Only screens fulfilling the pre-established quality criteria (Z’ factor > 0 and Spearman rank correlation between replicates > 0.7) were considered further. After data processing, potentially interesting siRNAs were selected based on a Z score > -1 in the KRAS WT and < -2 in the KRAS mutant cells.
(PDF)

S4 Fig Uncropped western blots from the main figures. (A) Fig 3A. (B) Fig 3D.
(PDF)

S5 Fig (A) Drug-dose response curves of PDAC cells after AZD5438 exposure in a fifteen-day colony formation assay. **P<0.01, ***P<0.001, ***P<0.0001, Two-way ANOVA. (B) Drug-dose response curves of CRC cells, *KRAS* WT/*BRAF* mutant (green) and *KRAS* WT/*BRAF* WT (black) cells after AZD5438 exposure in a five-day survival assay. ****P<0.0001, Two-way ANOVA. Error bars represent SEM of three technical replicates.
(PDF)

S6 Fig Uncropped western blots from the main figures. (A) Fig 6A. (B) Fig 6B.
(PDF)

S7 Fig Uncropped western blots from the main Fig 6C. (PDF)

S8 Fig Cell cycle profiles of SW48 KRAS WT and p.G12V cell lines after AZD5438 exposure. Propidium iodide (PI) flow cytometry plots. SW48 KRAS WT (A) and p.G12V (B) were exposed to 0.3 μM AZD5438 or DMSO for 16, 24 and 48 hours after which cell cycle profiles were assessed by flow cytometry. The KRAS p.G12V mutant cells showed a decrease in S and G_2_/M-phase cells after exposure with AZD5438 when compared to the control (DMSO) and to KRAS WT cells (AZD5438 and DMSO).
(PDF)

S9 Fig Uncropped western blots from the main figures. (A) Fig 6I. (B) Fig 6J.
(PDF)

S10 Fig Response to three second-generation CDK inhibitors in CRC cancer cell panel. (A) AT7519, (B) dinaciclib and (C) PD023309 survival curves from a five-day cell viability assay to assess the KRAS selectivity of the CDK inhibitors in ten colorectal cell lines, four KRAS WT (black) and six mutant (pink) cell lines.
(PDF)

S11 Fig (A and B) Average increase in tumour volume of KRAS WT and mutant xenografts. In the KRAS WT xenografts there is no significant difference between the treatment arm and the non-treatment. As in the KRAS mutant xenografts, the drugged arm shows significantly reduced tumour growth compared to the vehicle. Error bars represent SEM. (ns not-significant, **p < 0.01, non-paired t-test). (C) Average final tumour weight. There is no significant difference between the vehicle and treatment arms, however the difference in weight between the WT and mutant treated with AZD5438 is significant (ns not-significant, **p < 0.01, t-test).
(PDF)

S1 Table Results from the high throughput siRNA screen. This table lists the genes included in the siRNA library alongside the gene accession number and the median Z scores from three replicate screens for each cell line.
(XLSX)

S2 Table List of Colorectal and PDAC non-isogenic cell lines used in this study. (XLSX)

S3 Table Tables presenting SF_50_, and the area under the curve (AUC) of CDK1 inhibitors, RO-3306 and AZD5438, for SW48 KRAS isogenic cell lines (A), Colorectal (B) and Pancreatic Adenocarcinoma Cancer cell lines (C).
(XLSX)

S4 Table Tables presenting SF_50_ results, and the area under the curve (AUC), of the different CDK inhibitors, AT7519, dinaciclib and PD023309 in a panel of colorectal cell lines. (XLSX)
