# Correction: CDK1 Is a Synthetic Lethal Target for KRAS Mutant Tumours

**DOI:** 10.1371/journal.pone.0154007

**Published:** 2016-04-18

**Authors:** Sara Costa-Cabral, Rachel Brough, Asha Konde, Marieke Aarts, James Campbell, Eliana Marinari, Jenna Riffell, Alberto Bardelli, Christopher Torrance, Christopher J. Lord, Alan Ashworth

There is an error in S1 Table. Columns were incorrectly labeled and the data in columns N and R were erroneously duplicated. Please see the corrected S1 Table here.

## Supporting Information

S1 Table Results from the high throughput siRNA. This table lists the genes included in the siRNA library alongside the gene accession number and the median Z scores from three replicate screens for each cell line.
(XLSX)
