# Correction: CDK1 Is a Synthetic Lethal Target for KRAS Mutant Tumours

**DOI:** 10.1371/journal.pone.0176578

**Published:** 2017-04-20

**Authors:** Sara Costa-Cabral, Rachel Brough, Asha Konde, Marieke Aarts, James Campbell, Eliana Marinari, Jenna Riffell, Alberto Bardelli, Christopher Torrance, Christopher J. Lord, Alan Ashworth

The affiliation for the eighth author is incorrect. Alberto Bartelli is not affiliated with #3 but with #1 The CRUK Gene Function Laboratory, The Institute of Cancer Research, London, SW3 6JB, United Kingdom and #2 Breakthrough Breast Cancer Research Centre, The Institute of Cancer Research, London, SW3 6JB, United Kingdom.

## References

[1] Costa-Cabral S, Brough R, Konde A, Aarts M, Campbell J, Marinari E, et al (2016) CDK1 Is a Synthetic Lethal Target for KRAS Mutant Tumours. PLoS ONE 11(2): e0149099 doi: 10.1371/journal.pone.0149099 26881434 10.1371/journal.pone.0149099 PMC4755568

