# Correction: Correction: CDK1 Is a Synthetic Lethal Target for KRAS Mutant Tumours

**DOI:** 10.1371/journal.pone.0206729

**Published:** 2018-10-26

**Authors:** Sara Costa-Cabral, Rachel Brough, Asha Konde, Marieke Aarts, James Campbell, Eliana Marinari, Jenna Riffell, Alberto Bardelli, Christopher Torrance, Christopher J. Lord, Alan Ashworth

There is an error in the Correction published on April 20, 2017. The correct affiliation for the eighth author, Alberto Bartelli, is Candiolo Cancer Institute-FPO, IRCCS, Candiolo, TO, Italy and Department of Oncology, University of Torino, Candiolo, TO 10060, Italy.
