# Supplementary figures and images for: CDK1 Is a Synthetic Lethal Target for KRAS Mutant Tumours
Source: PLoS One. 2016 Feb 16;11(2):e0149099. doi: 10.1371/journal.pone.0149099 (PMC4755568; doi:10.1371/journal.pone.0149099)

# S1 Fig

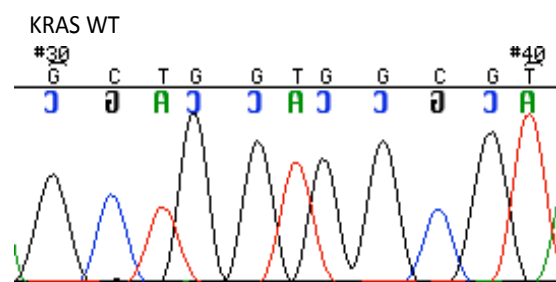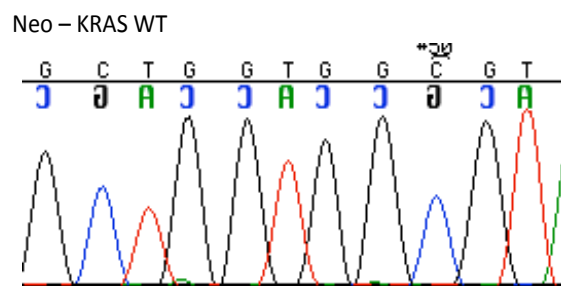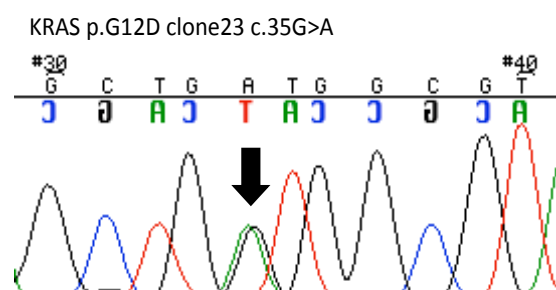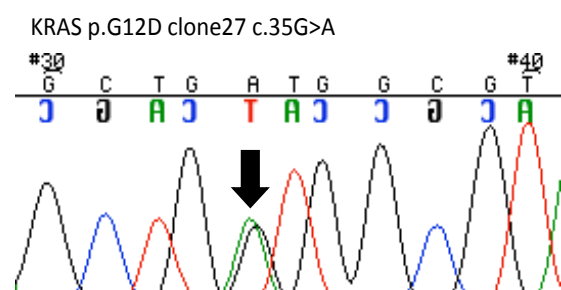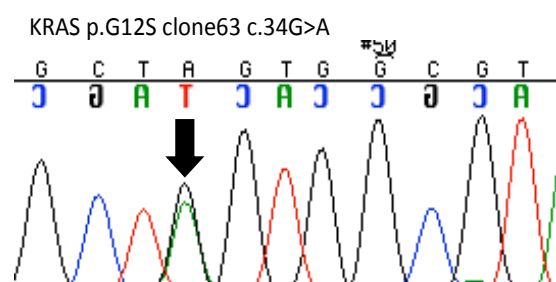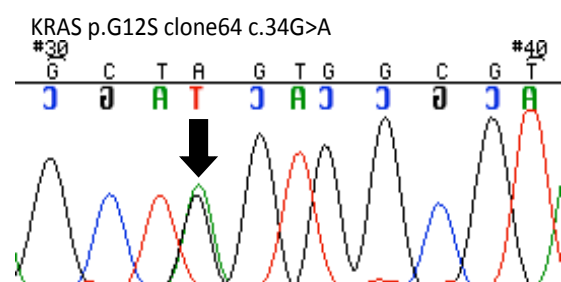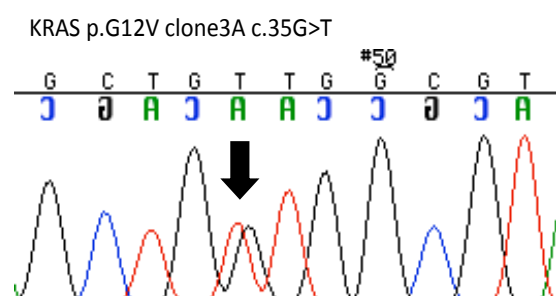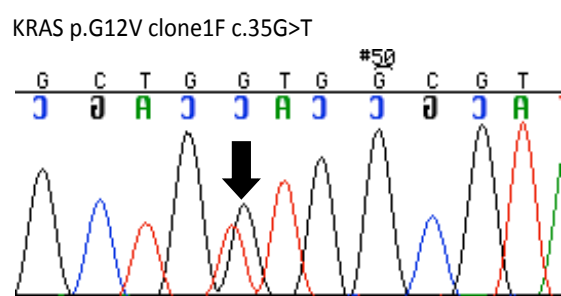

Supplement: S1 Fig — The introduced genetic alterations in the targeted cells was determined by RT-PCR and sequencing of the KRAS transcript. The different introduced mutations are indicated for each cell line with a black arrow in the chromatograms. (PDF) [file pone.0149099.s001.pdf]

S2 Fig

A. (see also Fig 1B)

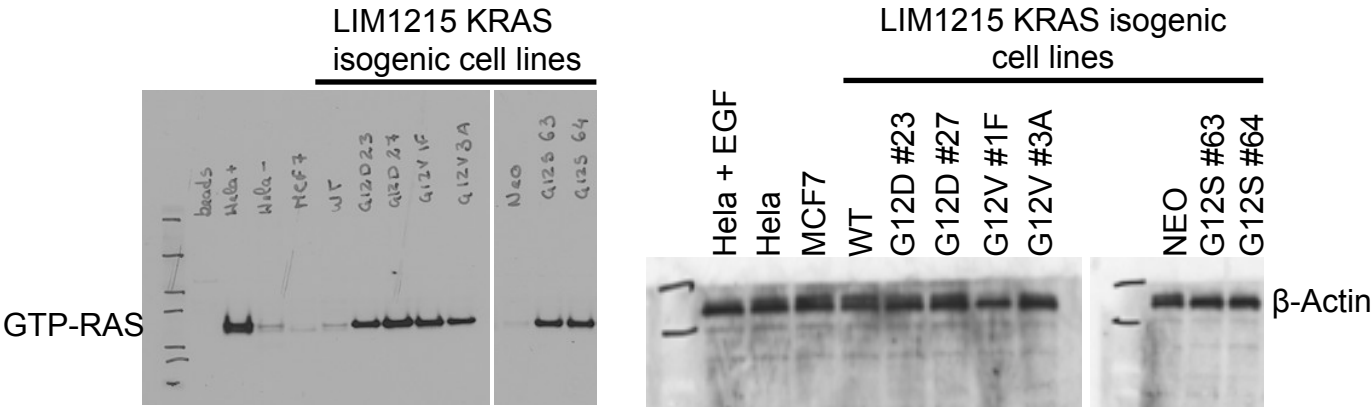

B. (see also Fig 1E)

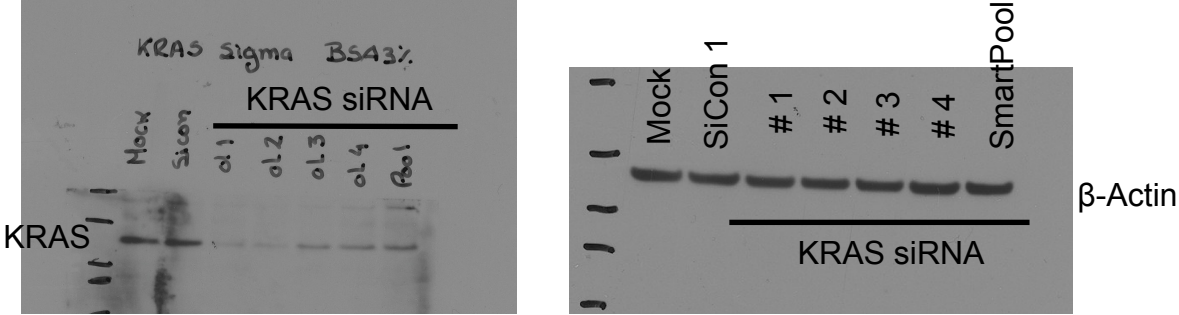

Supplement: S2 Fig — (A) Fig 1B. (B) Fig 1E. (PDF) [file pone.0149099.s002.pdf]

S3 Fig

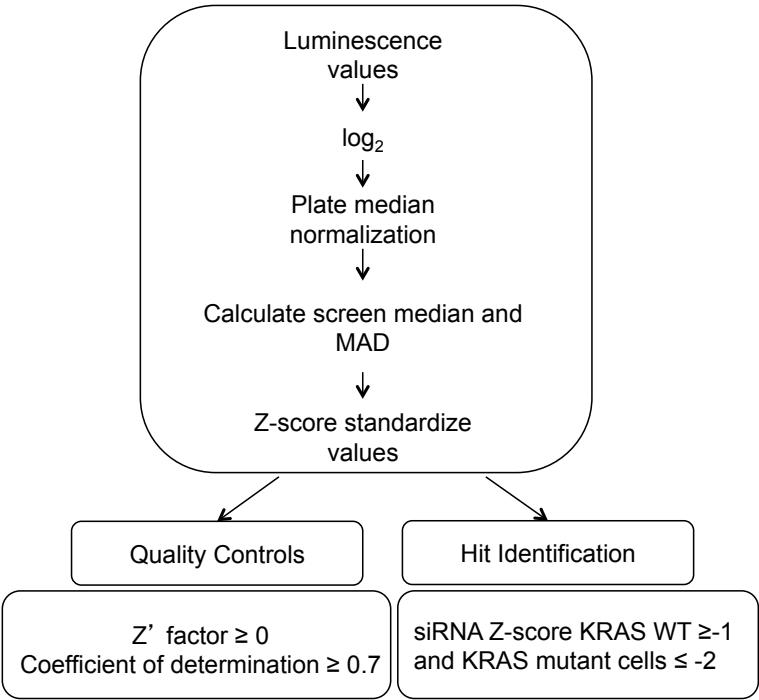

Supplement: S3 Fig — Cell viability was assessed at day six by CellTiter-Glo (Promega) luminescence reading. Only screens fulfilling the pre-established quality criteria (Z’ factor > 0 and Spearman rank correlation between replicates > 0.7) were considered further. After data processing, potentially interesting siRNAs were selected based on a Z score > -1 in the KRAS WT and < -2 in the KRAS mutant cells. (PDF) [file pone.0149099.s003.pdf]

S4 Fig

A. (see also Fig 3A)

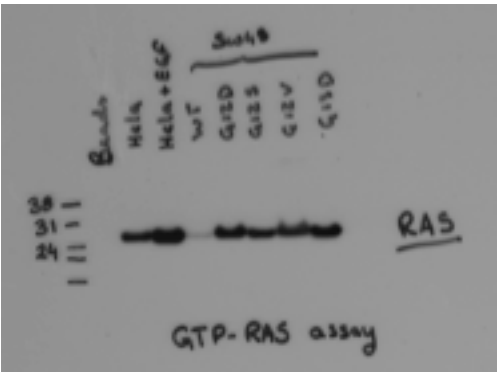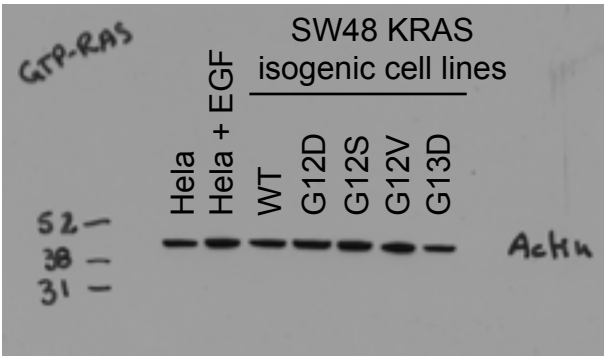

B. (see also Fig 3D)

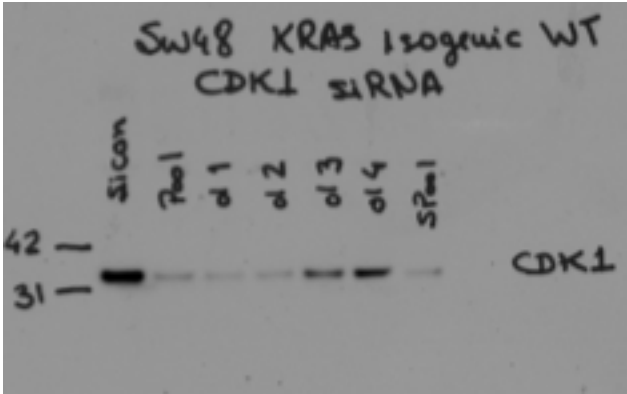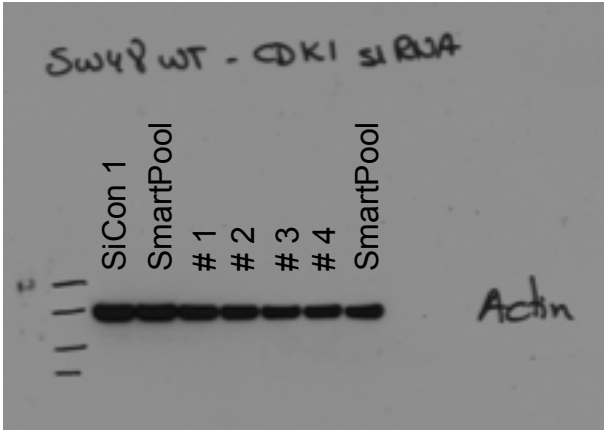

Supplement: S4 Fig — (A) Fig 3A. (B) Fig 3D. (PDF) [file pone.0149099.s004.pdf]

S5 Fig

A.

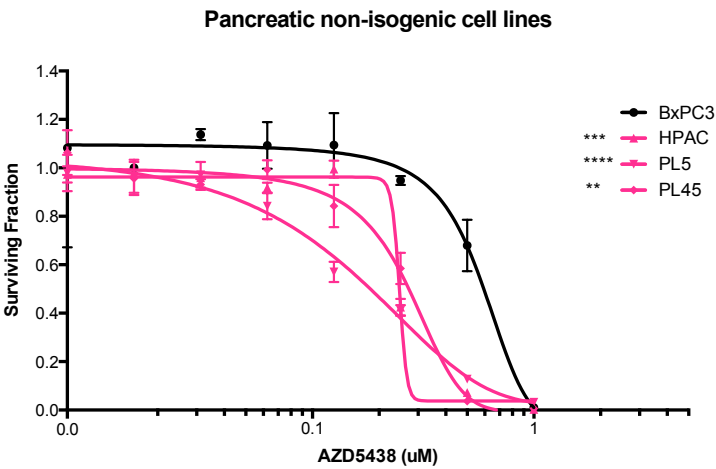

B.

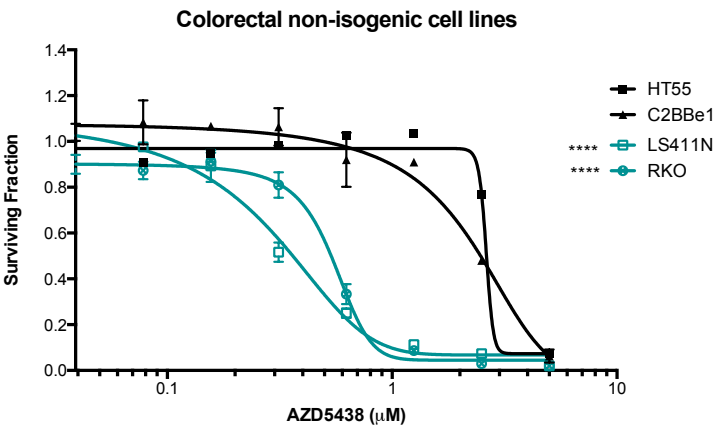

Supplement: S5 Fig — (A) Drug-dose response curves of PDAC cells after AZD5438 exposure in a fifteen-day colony formation assay. **P<0.01, ***P<0.001, ***P<0.0001, Two-way ANOVA. (B) Drug-dose response curves of CRC cells, KRAS WT/BRAF mutant (green) and KRAS WT/BRAF WT (black) cells after AZD5438 exposure in a five-day survival assay. ****P<0.0001, Two-way ANOVA. Error bars represent SEM of three technical replicates. (PDF) [file pone.0149099.s005.pdf]

S6 Fig

A. (see also Fig 6A)

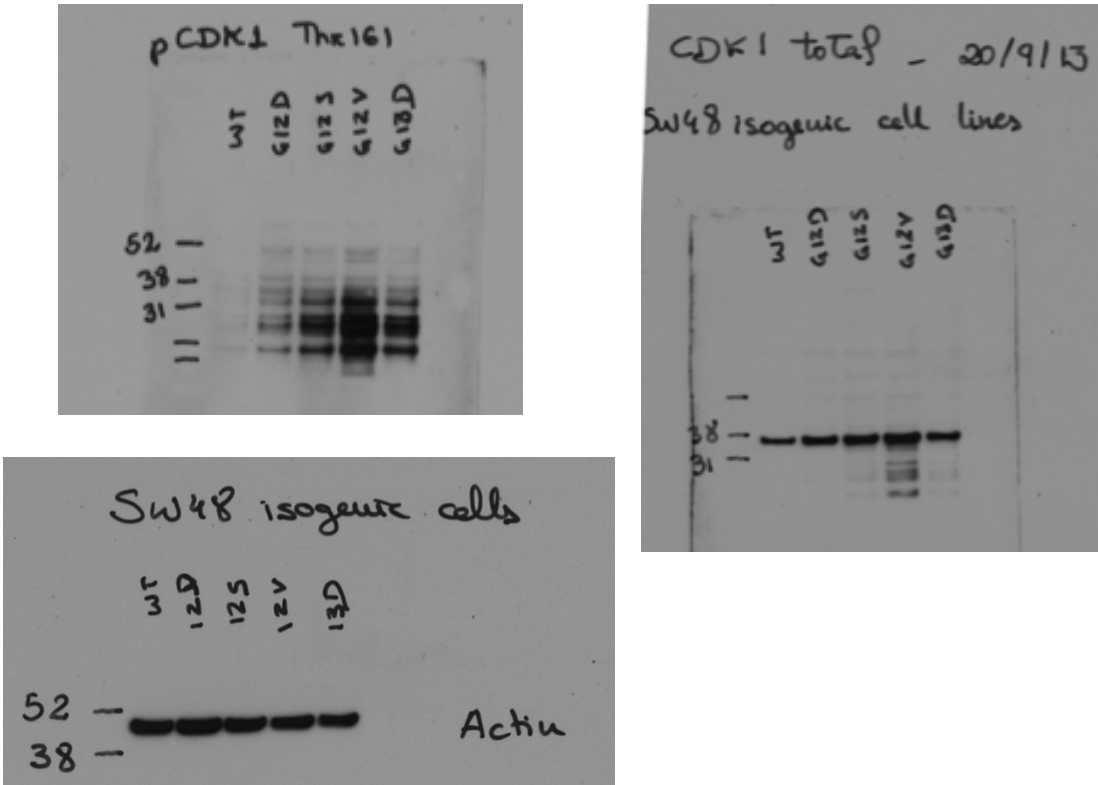

B. (see also Fig 6B)

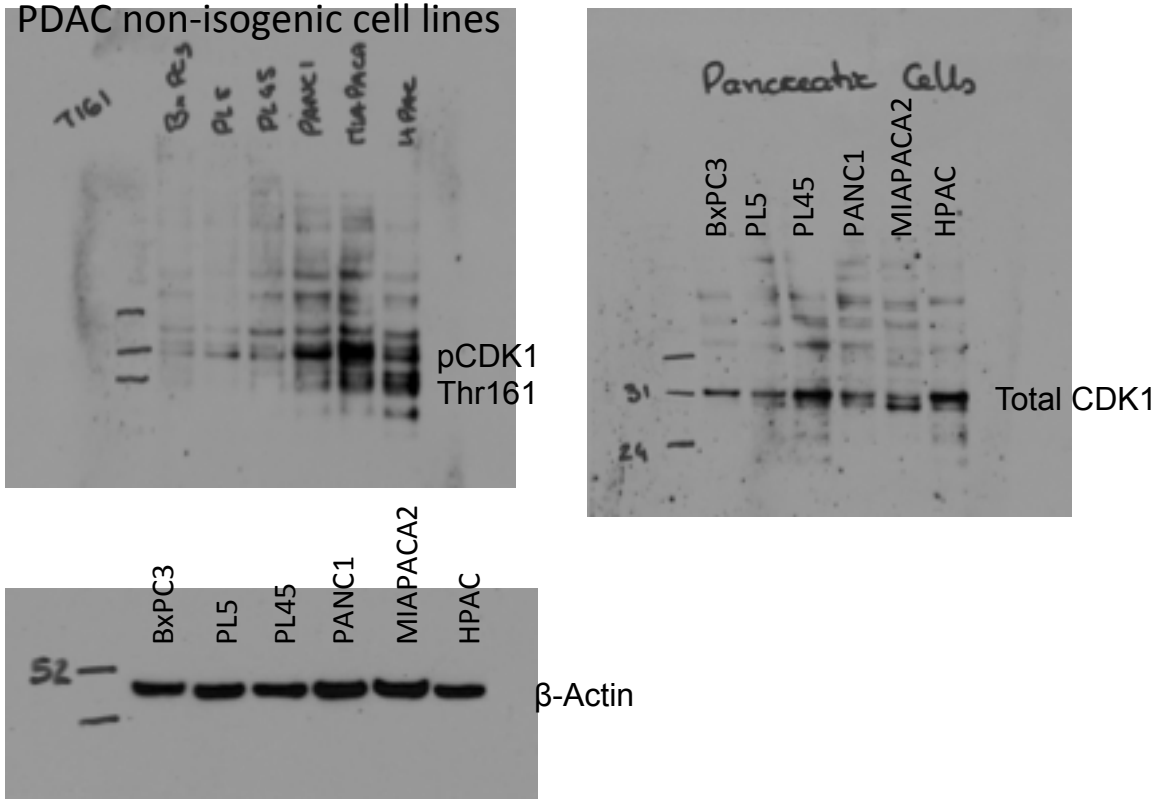

Supplement: S6 Fig — (A) Fig 6A. (B) Fig 6B. (PDF) [file pone.0149099.s006.pdf]

S7 Fig

(see also Fig 6C)

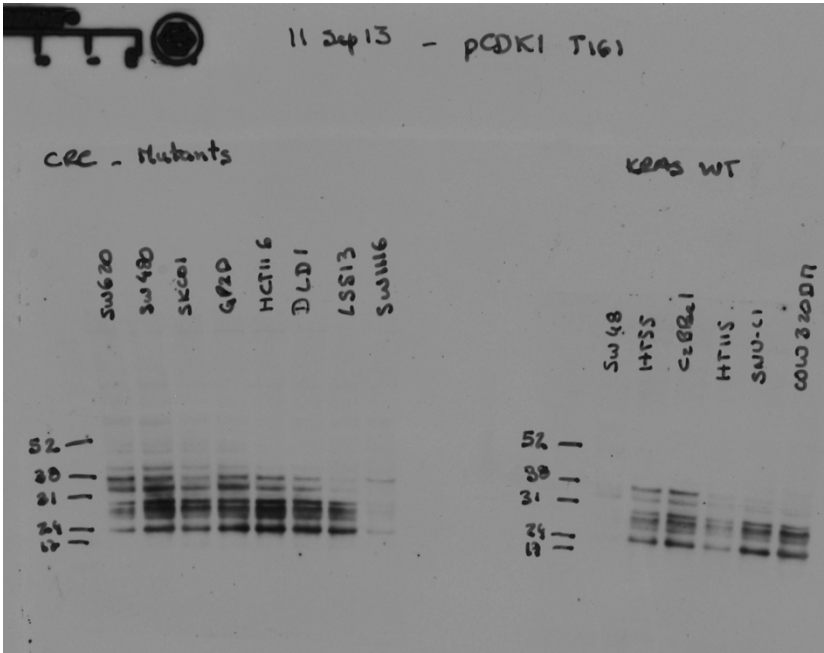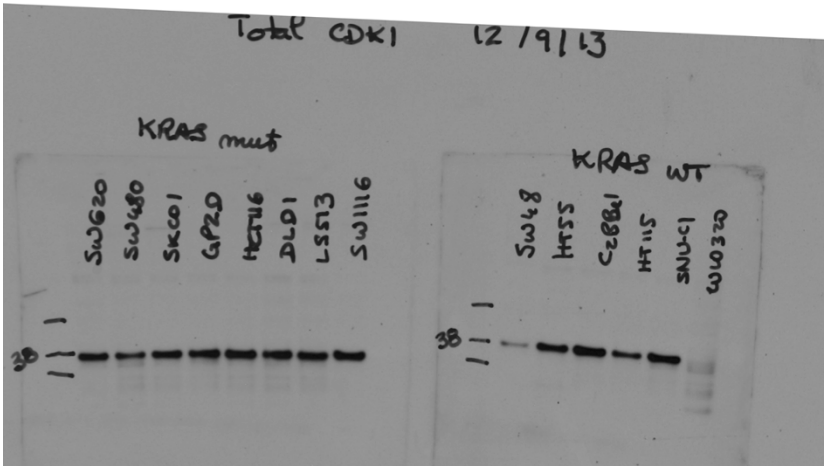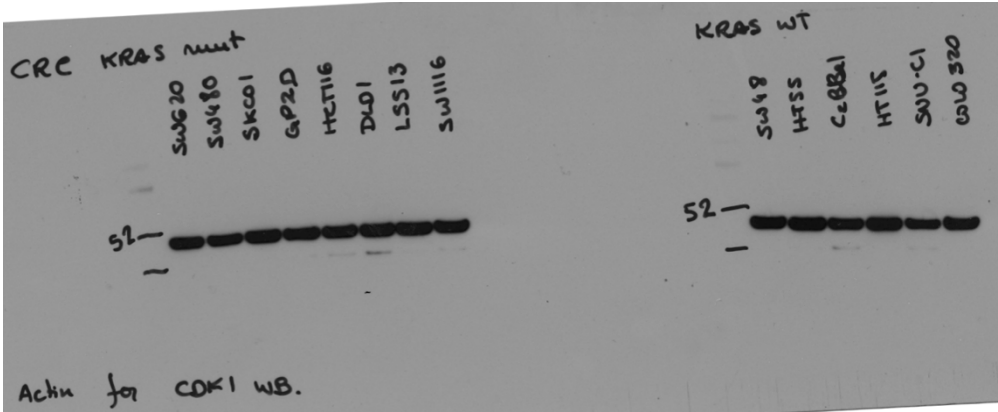

Supplement: S7 Fig — (PDF) [file pone.0149099.s007.pdf]

S8 Fig

A.

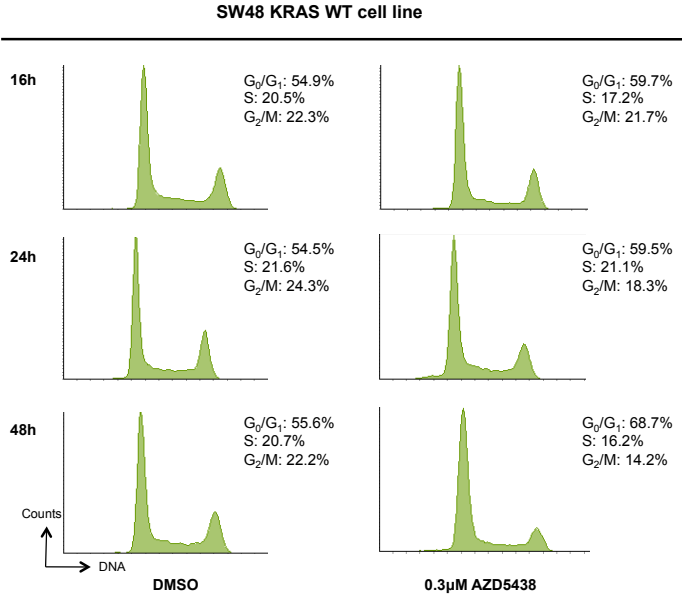

B.

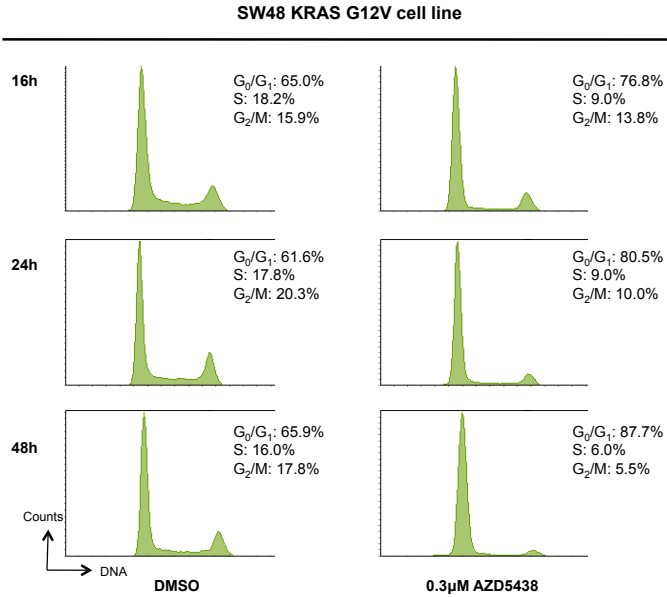

Supplement: S8 Fig — Propidium iodide (PI) flow cytometry plots. SW48 KRAS WT (A) and p.G12V (B) were exposed to 0.3 μM AZD5438 or DMSO for 16, 24 and 48 hours after which cell cycle profiles were assessed by flow cytometry. The KRAS p.G12V mutant cells showed a decrease in S and G2/M-phase cells after exposure with AZD5438 when compared to the control (DMSO) and to KRAS WT cells (AZD5438 and DMSO). (PDF) [file pone.0149099.s008.pdf]

S9 Fig

A. (see also Fig 6I)

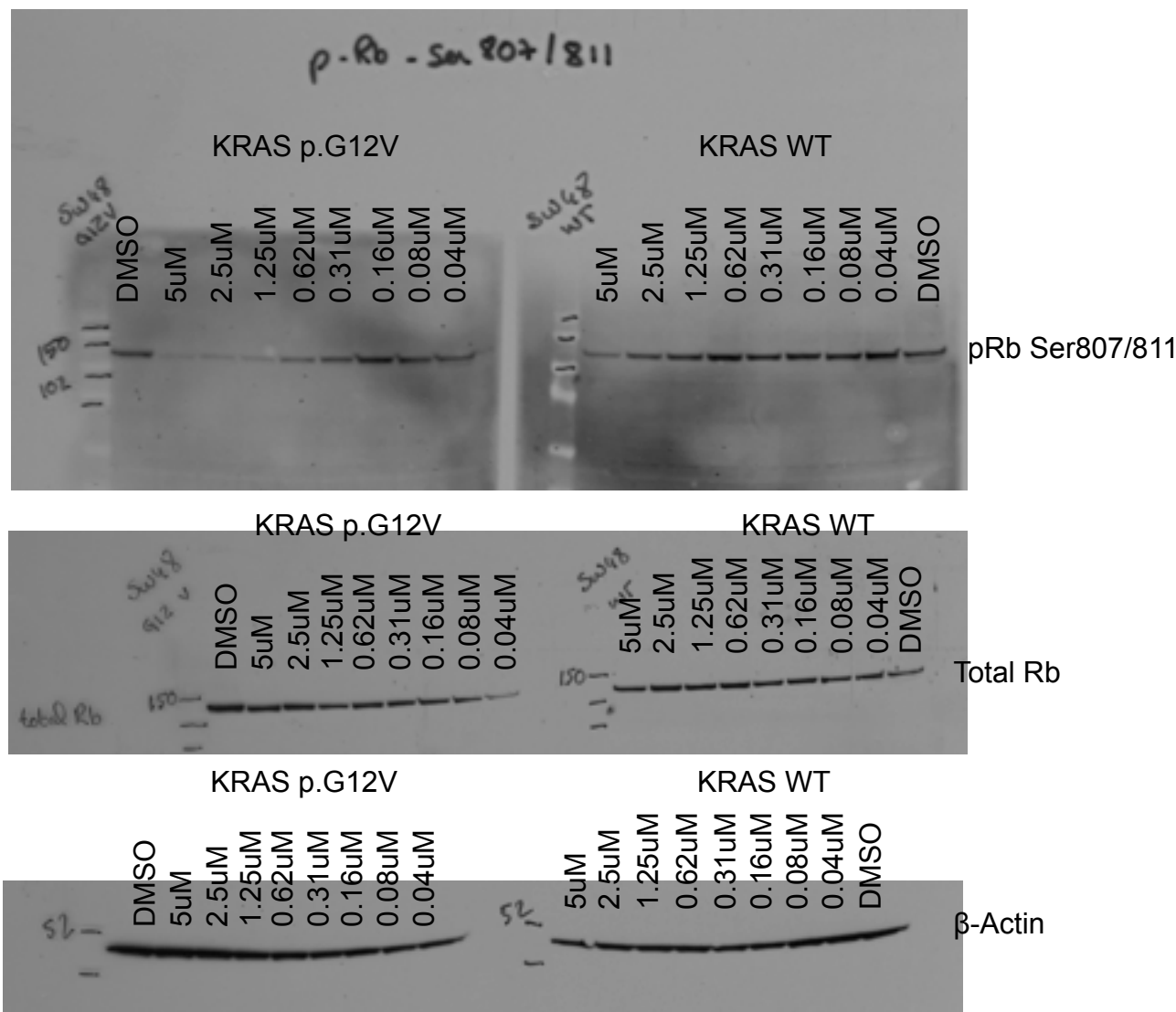

B. (see also Fig 6J)

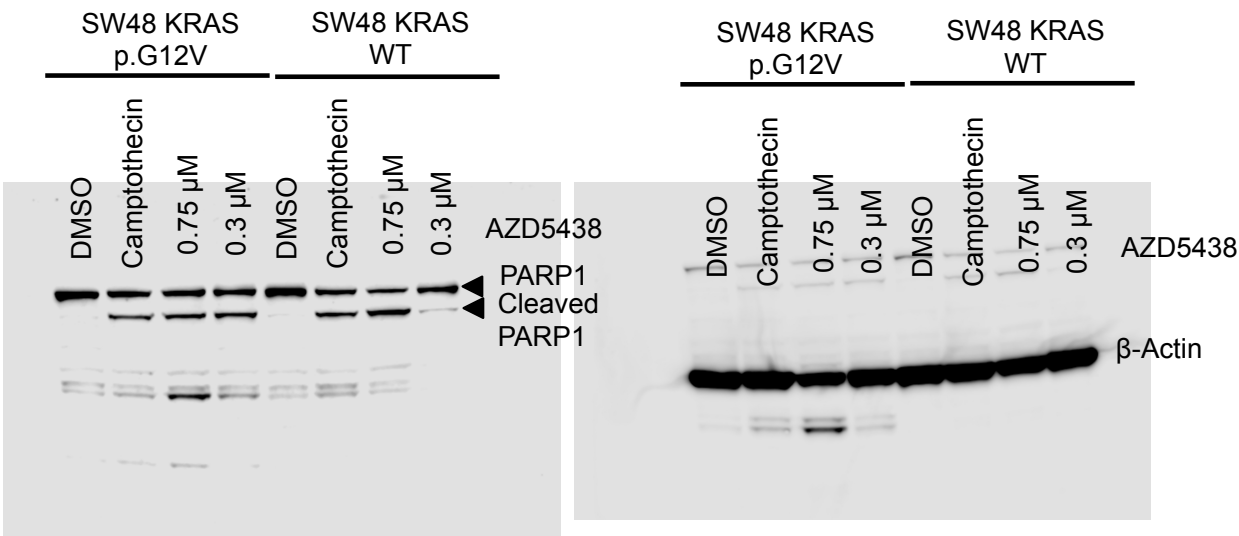

Supplement: S9 Fig — (A) Fig 6I. (B) Fig 6J. (PDF) [file pone.0149099.s009.pdf]

A.

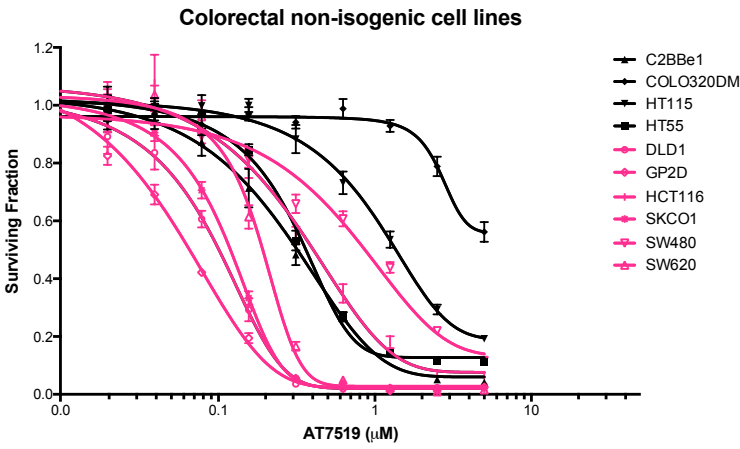

B.

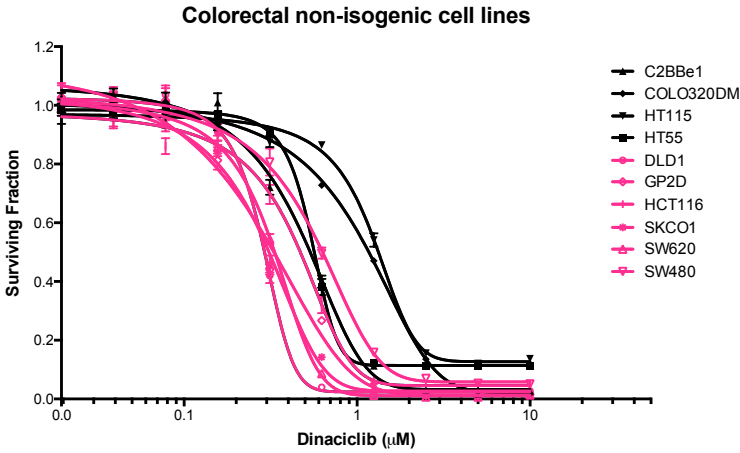

C.

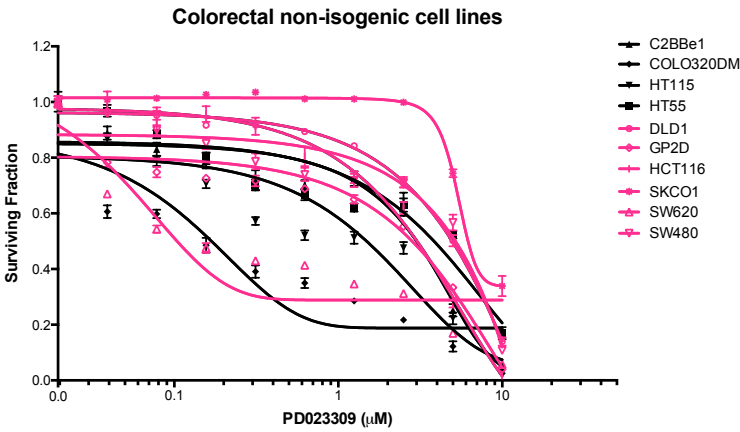

Supplement: S10 Fig — (A) AT7519, (B) dinaciclib and (C) PD023309 survival curves from a five-day cell viability assay to assess the KRAS selectivity of the CDK inhibitors in ten colorectal cell lines, four KRAS WT (black) and six mutant (pink) cell lines. (PDF) [file pone.0149099.s010.pdf]

S11 Fig

A.

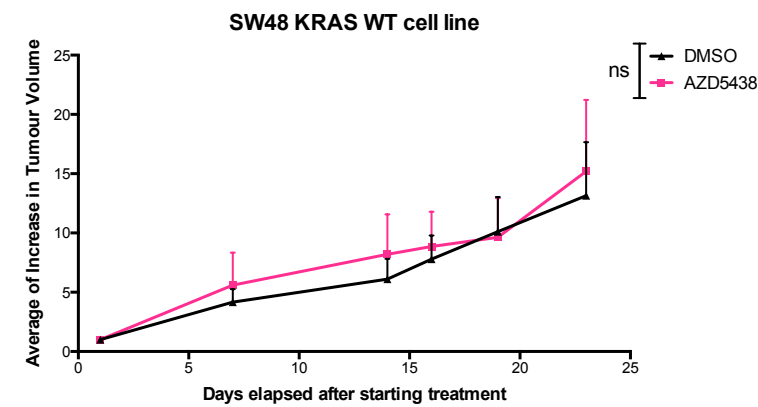

B.

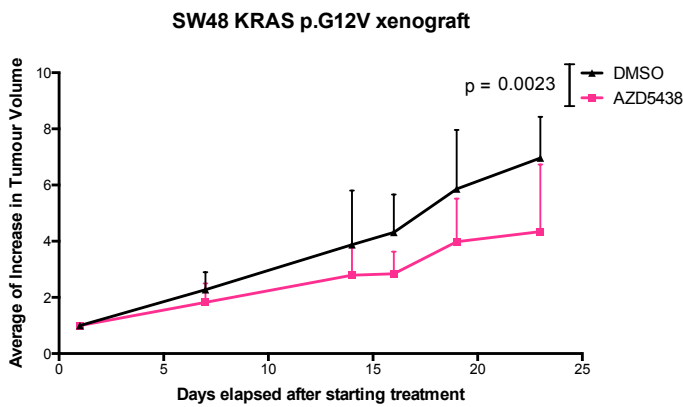

C.

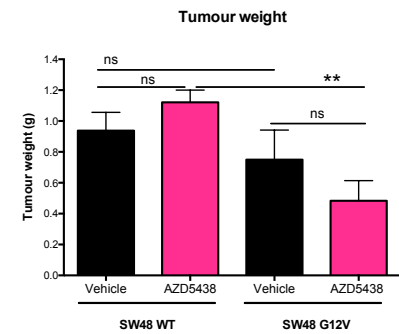

Supplement: S11 Fig — (A and B) Average increase in tumour volume of KRAS WT and mutant xenografts. In the KRAS WT xenografts there is no significant difference between the treatment arm and the non-treatment. As in the KRAS mutant xenografts, the drugged arm shows significantly reduced tumour growth compared to the vehicle. Error bars represent SEM. (ns not-significant, **p < 0.01, non-paired t-test). (C) Average final tumour weight. There is no significant difference between the vehicle and treatment arms, however the difference in weight between the WT and mutant treated with AZD5438 is significant (ns not-significant, **p < 0.01, t-test). (PDF) [file pone.0149099.s011.pdf]
